# Adaptive–based cascaded PI–PID controller for load frequency regulation with real time verification

**DOI:** 10.1371/journal.pone.0362396

**Published:** 2026-11-06

**Authors:** Abdullah M. Alharbi, Mohamed A. Awad, Mahmoud A. Attia, S.F. Mekhamer, Ahmed H. EL-Ebiary

**Affiliations:** 1 Department of Electrical Engineering, College of Engineering at Wadi Addawasir, Prince Sattam bin, Abdulaziz University, Wadi Addawasir, Saudi Arabia; 2 Department of Electrical Power & Machines, Faculty of Engineering, Ain Shams University, Cairo, Egypt; 3 Faculty of Engineering Technology, ElSewedy University of Technology, Cairo, Egypt; 4 Electrical Engineering Department, Faculty of Engineering, Future University in Egypt, New Cairo, Egypt; Netaji Subhas University of Technology Dwarka, INDIA

## Abstract

In order to address Load Frequency Control (LFC) problems in single-area and interconnected two-area power systems, this work proposes a cascaded control scheme that combines PI–PID control with the Set-Membership Affine Projection Algorithm (SMAPA). The proposed approach is evaluated under various practical operating conditions, including step load disturbances, random load variations, wind power fluctuations, and parameter uncertainty. In addition, frequency-domain stability analysis is performed to further assess the robustness of the proposed controller, while real-time validation is conducted for the two-area system to demonstrate its practical effectiveness. The proposed controller is compared with conventional PI controllers tuned using five optimization methods for the single-area system. For the two-area system, its performance is benchmarked against PID controllers designed using three optimization methods and the recently reported SPPI-based approach. The simulation results show that the proposed controller achieves lower overshoot and faster settling time in both configurations. These improvements are attributed to the integration of real-time adaptive parameter updating provided by SMAPA with the favorable dynamic characteristics of the PI–PID control structure. The results demonstrate that the proposed cascaded SMAPA-based PI–PID controller provides robust frequency regulation under different operating conditions and offers promising potential for practical interconnected power system applications.

## 1. Introduction

Maintaining the power system frequency at its nominal level is the main objective of load frequency control rules in linked area power systems. Changes in load demand led to frequency variation in all linked areas. This calls for the employment of an efficient controller that can return frequency signal to nominal value. The area control error (ACE), which is the total of each area's frequency deviation and tie line power deviation, is fed into LFC controllers. The controller generates a corrective signal that adjusts the governor setpoint of generators in each area, Consequently, the generated power of each generator is updated and then the system frequency is restored to their normal.

This section reviews various control topologies, modeling configurations, and optimization techniques applied in LFC studies. Due to their ease of use and efficiency, classical controllers like (PI) and (PID) controllers have been widely utilized. For example, in [1] a particle swarm optimization (PSO) based PI controller was applied to a dual-area power system with varies generation sources subjected to step load perturbations. In [2] a conventional PID controller optimized by Genetic Algorithm in interconnected two area system was used. Similarly, in [3], a two-area hydrothermal system was subjected to a PID controller that was tuned using ant colony optimization (ACO), taking into consideration nonlinear constraints such governor dead band and generation rate constraint (GRC) under various cost functions.

Advanced intelligent and adaptive control strategies have also been explored. In [4] A multilayer perceptron neural network (MLPNN) controller modified by reinforcement learning outperformed a bacterial foraging-based integral controller (BFIC) when evaluated under significant load variations and step disturbances. Fractional order PI (FOPI) and fractional order PID (FOPID) controllers showed a better performance than integer-order controllers when applied to both single and interconnected systems in [5]. In [6], a hybrid power system consisting of diesel generators, fuel cells, photovoltaic units, wind turbines, and different energy storage systems was equipped with an adaptive fuzzy PID (AFPID) controller tunned using an improved sine cosine algorithm (ISCA). When compared to traditional PID control, simulation results showed improved stability and quicker dynamic response.

Robust control techniques have also been investigated. In [7], a sliding mode controller (SMC) was exposed to a multi-area time-delay hybrid system and tested under varying wind penetration levels, load disturbances, and parameter variations, outperforming conventional PI controllers. In [8] a PSO-based H-infinity controller was proposed for control of frequency for single hybrid distributed generation system and compared with GA-based H-infinity and droop-based controllers. Additionally, the study presented the application of different energy storage elements to lower frequency deviations.

In [9], an improved artificial bee colony pso (IABC-PSO) method was adopted to improve a cascaded (1 + PI)–PI–PID controller for a two-area hydrothermal system that included capacitive energy storage (CES). The controller was tested under step load perturbations and parameter variations, demonstrating robust and efficient dynamic performance.

Various system configurations have been considered in LFC studies like single-area systems [10], double-area systems [11], three-area systems [12], four-area systems [13], and large-scale multi-area systems [14]. To enhance modeling accuracy and realism, additional components and technologies have been incorporated. For instance, flexible ac transmission system (FACTS) devices were studied in [15], energy storage systems in [16], and electric vehicles along with HVDC interconnections in [17], particularly within primary frequency control frameworks to minimize frequency deviations among interconnected areas.

Recent studies have focused on adaptive, intelligent, and data-driven techniques to improve LFC performance under changing operating conditions. In [18], a data-driven adaptive predictive frequency control method was proposed for multi-area power systems with unknown and time-varying inertia. The method adaptively updates a data-based system representation using historical input–output data and moving-horizon estimation, followed by predictive control optimization. However, the use of data-based modeling and online optimization results in a more complex control framework than conventional PI/PID-based adaptive control.

In [19], an artificial-intelligence-based multistage controller was developed for a two-area power system with renewable generation. The proposed controller combines a multistage control structure with a metaheuristic optimization algorithm and was tested under load variations, parameter variations, and nonlinearities. Although the method achieved improved dynamic performance, its multistage structure and optimization-based tuning increase the complexity of the control design.

More recently, in [20], an HS-optimized Single Perceptron PI (SPPI) controller was proposed for single-area LFC, while a cascaded SPPI–PID structure was developed for a two-area system under renewable-energy uncertainty and time delays. The study also considered random load variations, wind disturbances, parameter variations, frequency-domain stability, and real-time implementation. However, its online adaptive mechanism is based on a single-perceptron formulation. In contrast, the present study employs SMAPA as the online adaptive mechanism within the cascaded PI–PID structure.

Although these recent studies demonstrate the effectiveness of adaptive, intelligent, and data-driven approaches for LFC, they use different control structures and adaptation mechanisms. Data-driven predictive methods require data-based modeling and optimization, while intelligent multistage approaches may involve more complex controller structures. The recent SPPI-based approach provides a simple adaptive solution, but it uses a single-perceptron mechanism. Therefore, this study investigates SMAPA as an alternative online adaptive mechanism within a cascaded PI–PID structure. The proposed controller is evaluated under step, random, and wind disturbances, parameter uncertainty, frequency-domain stability conditions, and real-time implementation, and is directly compared with the SPPI approach.

## 2. Contribution

An adaptive cascaded SMAPA–PID controller is proposed for load frequency control (LFC) of single-area and two-area interconnected power systems under different load and renewable-energy disturbances. Unlike conventional fixed-gain PI/PID controllers, the proposed approach uses the Set Membership Affine Projection Algorithm (SMAPA) as an online adaptive mechanism to continuously adjust the proportional and integral control parameters according to real-time control error information. This enables the controller to adapt its control action to changes in system operating conditions without relying only on fixed controller parameters determined offline.

The main contributions of this study are summarized as follows:

1. **Online adaptive SMAPA–PID control:** An adaptive cascaded SMAPA–PID control architecture is developed in which the SMAPA mechanism is integrated with the PID-based control structure to provide online adjustment of the controller parameters according to real-time area control error (ACE).
2. **Adaptive response under different disturbances:** The proposed controller is evaluated under step load changes, random load variations, and wind-energy-based disturbances in both single-area and two-area interconnected LFC systems.
3. **Integration of adaptive and cascaded control:** The proposed framework combines the online adaptation capability of SMAPA with the established PID control structure to improve transient frequency response while reducing the dependence on fixed controller gains.
4. **Parameter uncertainty and stability analysis:** The robustness of the proposed controller is evaluated under parameter uncertainty through variations in the main system parameters. In addition, frequency-domain analysis is performed to assess the stability and robustness of the proposed control strategy.
5. **Comparison with existing controllers:** The proposed controller is compared with conventional PI/PID controllers and the recently reported SPPI controller using quantitative performance measures, including frequency deviation, overshoot/undershoot, settling time, and integral performance indices.
6. **Real-time validation:** The feasibility of the proposed adaptive control strategy is further investigated through real-time implementation, providing practical validation in addition to simulation-based results.

The proposed framework therefore provides an adaptive cascaded control strategy for LFC that can dynamically respond to changes in load and renewable-energy operating conditions while retaining a relatively simple PID-based control structure.

## 3. System model

### 3.1. Single area system

Fig 1 presented the modeling of single area under study. A load change, ∆P_L_, enters the system as a disturbance at the generator load interface. The secondary controller takes the observed frequency deviation and mixes it with the primary droop control signal before entering it into the block of governor element. The governor and turbine take this combined control signal and extract a mechanical power to overcome the error in the frequency. The secondary completes the closed-loop dynamic response and ensures the elimination of frequency error. The governor adjusts the valve based on the control signal, then the turbine turns that valve movement into power, with some time delay in both steps this delay is expressed by an exponential mathematical representation with a time constant parameter of 2 second. The transfer function models of the system components are presented equations (1), (2), (3), (4), (5), (6). The system model variables are expressed in Table 1 [21].

**Table 1:** Values of System elements.

${\;\tau }_{gov}$	Governor system time constant	0.1 s
${\;\tau }_{\text{tur}}$	Steam turbine time constant	0.3 s
M	Inertia constant	10
D	Damping coefficient	1
R	Speed droop	0.05
B	Frequency bias factor	0.5
ρ	Air density	1.225
$C_{p}$	Power coefficient	0.5
Λ	Swept wind turbine area	*5538.96*
𝓋	Wind speed	

**Fig 1:**
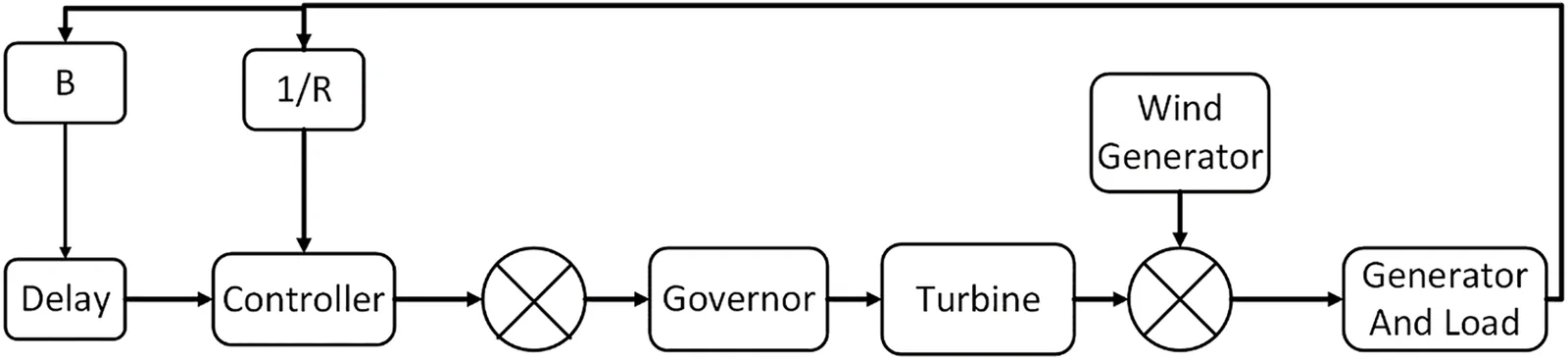
Single area power system modeling.


$$\begin{array}{c} Governor\;transfer\;function=\frac{\text{1}}{\text{1}+{S\;\tau }_{gov}} \end{array}$$ (1)

$$\begin{array}{c} Turbine\;transfer\;function=\frac{\text{1}}{\text{1}+{S\;\tau }_{tur}} \end{array}$$ (2)

$$\begin{array}{c} genrator\;and\;load\;transfer\;function=\frac{\text{1}}{MS+D} \end{array}$$ (3)

$$\begin{array}{c} droop=\frac{\text{1}}{R} \end{array}$$ (4)

$$\begin{array}{c} governor\;frequency\;bias=\frac{\text{1}}{B} \end{array}$$ (5)

$$\begin{array}{c} power\;from\;wind\;generator=\frac{\text{1}}{\text{2}}*\rho *\text{\textasciitilde{}\textasciitilde{}}\;C_{p}\;*\Lambda *\text{\textasciitilde{}\{𝓋}{\}}^{\text{3}} \end{array}$$ (6)

Where R is the governor's speed droop, The inertia constant is denoted by M, and Governor frequency bias is denoted by B.

### 3.2. Double area system

The system studied comprises two interconnected areas coupled by a tie-line that facilitates power exchange between them [22], as shown in Fig 2. The error of each area is fed into the controller that corresponds to it. Improving the system's transient response to load perturbations is the main goal. During steady state case, the controllers remain inactive as long as the frequency signal is kept at its normal value without deviations.

**Fig 2:**
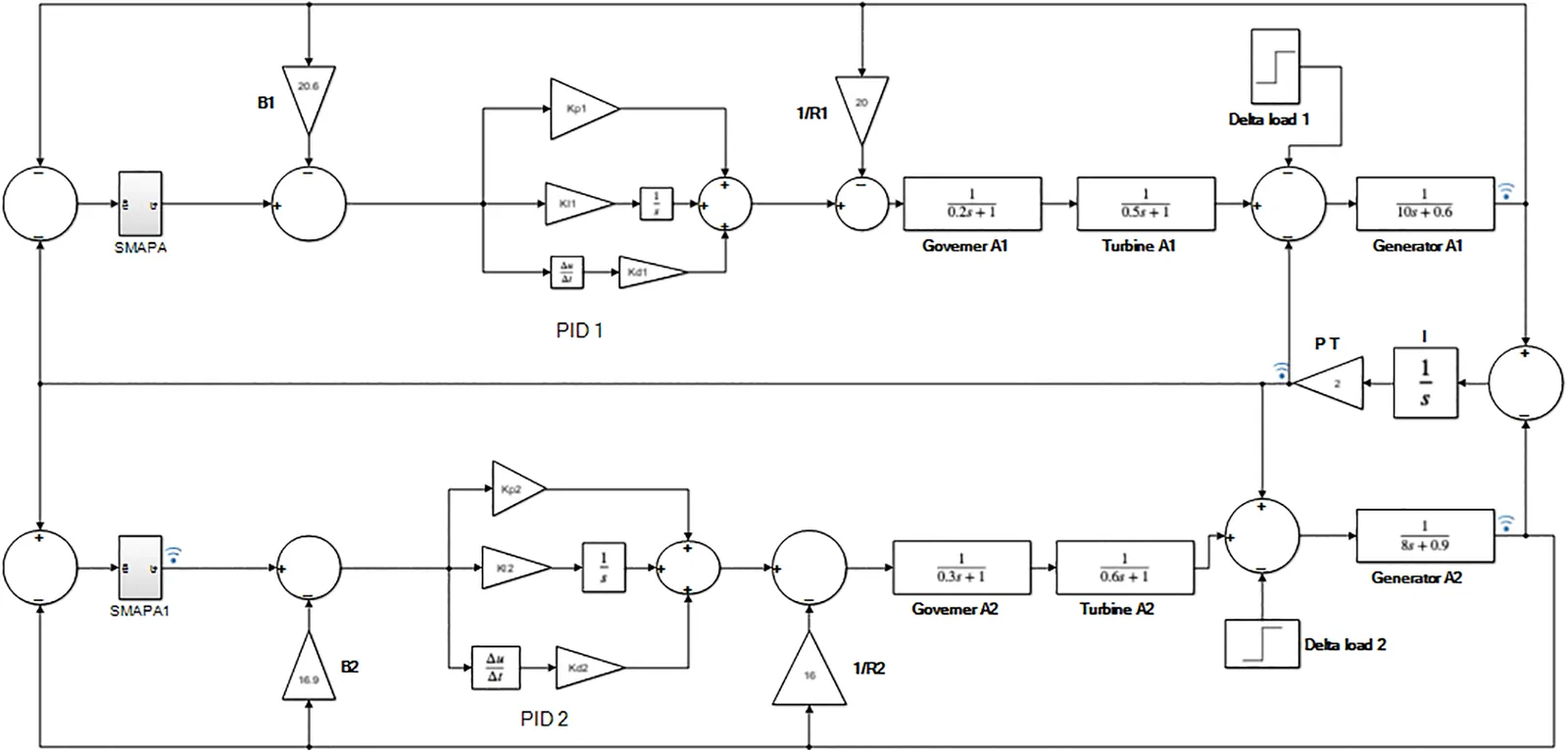
Two area power system model.

## 4. The proposed control strategy

### 4.1. PID controller

PID controllers are widely utilized in industrial closed-loop control systems. They generate control action based on the signal which is the difference between the measured value and the desired value. The controller parameters are carefully tuned to ensure robust closed-loop performance, as PID control enhances dynamic response while minimizing steady-state error [23]. The controller gains $\left(\kappa_{porpotional},\kappa_{integral},\kappa_{derivative}\right)\;$are obtained through system modeling and simulation using MATLAB. The proportional gain $\kappa_{porpotional}$ primarily reduces the rise time, the derivative gain $\kappa_{derivative}\;$suppresses overshoot and shortens the settling time, and the integral gain $\kappa_{integral}\;$effectively cancels steady-state error. PID control system is formulated in Equations (7), (8), (9).


$$\begin{array}{c} {Proptional}_{out}=\kappa_{porpotional}\;error(t) \end{array}$$ (7)

$$\begin{array}{c} {Integral}_{out}=\kappa_{integral}\int_{\text{0}}^{t}\;error(t) \end{array}$$ (8)

$$\begin{array}{c} {Derivative}_{out}=\kappa_{derivative}\frac{d}{dt}\;error(t) \end{array}$$ (9)

where $\kappa_{porpotional}$ denotes the proportional coefficient, $\kappa_{integral}$ represents the integral coefficient, and $\kappa_{derivative}\;$corresponds to the derivative coefficient. Equation (10) represents the mathematical description of the PID controller.


$$\begin{array}{c} G_{PID}=\kappa_{porpotional}+\frac{\kappa_{integral}}{S}+\kappa_{derivative}S\; \end{array}$$ (10)

### 4.2. Set membership affine projection algorithm (SMAPA) controller

Due to their reliance on fixed parameter values, conventional control systems are less able to adapt to variations brought on by shifts in load requirement and the stochastic character of non-polluting power resources. Such variations have a significant effect on system frequency in modern interconnected power systems, which lowers the effectiveness of conventional controllers. Consequently, this shortcoming has led to increased interest in online adaptive control schemes, which dynamically adjust their parameters using real-time error information to achieve improved steadiness and enhanced transient response with diverse load and non-polluting energy states [24–26]. The proportional and integral gains of the PI controller are adjusted online using the SMAPA, an adaptive control method [27,28]. As seen in Fig 3, this strategy attempts to lower area control error ACE to lower frequency deviation.

**Fig 3:**
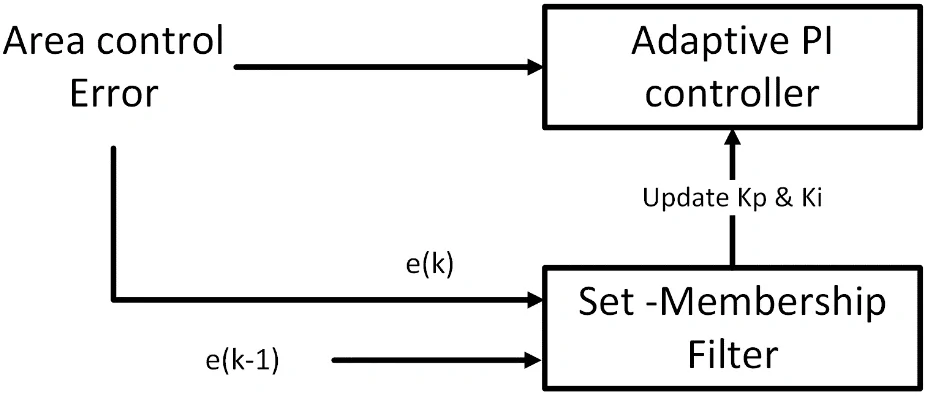
Shows the schematic structure of the SMAPA controller.


$$\begin{array}{c} X(i)={\left[\text{Error}(\text{i})-\text{Error}(\text{i}-\text{1}),\text{Error}(\text{i})+\text{Error}(\text{i}-\text{1})\right]}^{T} \end{array}$$ (11)

where Error(i) denotes the current control error, Error(i−1) is the control error at the previous sampling instant, and.X(i) represents the input vector of the SMAPA adaptive mechanism

𝓌(i) vector which describes the updated coefficients of the PI controller is described in equation (12).


$$\begin{array}{c} \text{𝓌}(i)={\left[\kappa_{porpotional},\kappa_{integral}\;\right]}^{T} \end{array}$$ (12)

where $\kappa_{porpotional}\;$and $\kappa_{integral}$ denote the adaptive proportional and integral weights, respectively. These weights determine the proportional and integral gains of the adaptive PI controller.

The change in the controller output between two consecutive sampling instants is defined as


$$\begin{array}{c} \Delta ⊔(i)=⊔(i)-⊔(i-\text{1}) \end{array}$$ (13)

Where ⊔~(i) denotes the current controller output and ⊔(i−1) represents the controller output at the previous sampling instant. Thus, Δ⊔(i) represents the actual change in the controller output.

The predicted change in the controller output is calculated as


$$\begin{array}{c} \hat{\Delta ⊔}(i)={\text{𝓌}(i-\text{1})}^{T}.X(i) \end{array}$$ (14)

where $\Delta \hat{⊔}(i)\;$denotes the predicted change in the controller output, 𝓌(i−1)  is the adaptive weight vector from the previous sampling instant, ${\text{𝓌}(i-\text{1})}^{T}\;$is its transpose, and X(i) is the current input vector.

The prediction error is then calculated as


$$\begin{array}{c} \text{£}(i)=\;\Delta ⊔(i)-\Delta \hat{⊔}(i) \end{array}$$ (15)

where £(i) denotes the prediction error between the actual and predicted changes in the controller output.

The adaptive weight vector is updated according to


$$\begin{array}{c} \text{\{𝓌}\}(i+\text{1})=\text{\textasciitilde{}}\text{𝓌}(i)+X(i){\left(X^{T}(i)\;X(i)\right)}^{-\text{1}}\alpha (i)\text{ £}(i) \end{array}$$ (16)

where 𝓌(i+1) represents the updated adaptive weight vector, $X^{T}\;$denotes the transpose of the input vector, represents the inverse of the input-vector energy term, α(i) is the adaptation factor, £(i) and is the prediction error

The adaptation factor is determined using the set-membership criterion as


$$\begin{array}{c} \alpha (i)=\left\{ \begin{array}{c} \text{1}-(\gamma /\left|\text{£}(i)\;\right|)\\ \;zero \end{array}\right.\;if\;\left\{ \begin{array}{c} \left|\text{£}(i)\right|>\gamma \\ otherwise \end{array}\right. \end{array}$$ (17)

where γ denotes the prescribed error tolerance or set membership bound. When the absolute prediction error exceeds γ, the adaptive factor becomes positive, and the controller weights are updated according to (16). When the prediction error remains within the prescribed tolerance α(i)=0 and the adaptive weights remain unchanged.

The adaptive PI gains are subsequently obtained from the updated weights as


$$\begin{array}{c} K_{p}(i)=\kappa_{porpotional}(i)\; \end{array}$$ (18)

$$\begin{array}{c} K_{i}(i)=\text{max}\left(\kappa_{integral}(i),\text{0}\right)\; \end{array}$$ (19)

where $K_{p}(i)\;$and $K_{i}(i)\;$denote the adaptive proportional and integral gains, respectively. The non-negative constraint on $K_{i}(i)\;$prevents the integral gain from taking a negative value.

The main steps of the proposed SMAPA-based adaptive PI controller are summarized in Algorithm 1 to clearly illustrate the online parameter adaptation procedure.

Algorithm 1 Pesudo Code for SMAPA

1. Construct the input vector:

  X(i) = [e(i)−e(i− 1), e(i)+e(i − 1)]ᵀ

2. Compute the controller-output change:

  ΔU(i) = U(i) − U(i−1)

3. Predict the output change:

  ΔÛ(i) = wᵀ(i−1) X(i)

4. Compute the prediction error:

  ε(i) = ΔU(i) − ΔÛ(i)

5. If |ε(i)| > γ then

  α(i) = 1 − γ/|ε(i)|

  w(i) = w(i−1) +

  X(i)[Xᵀ(i)X(i)]^−1^ α(i)ε(i)

Else

  w(i) = w(i−1)

End If

6. Update the adaptive gains:

  Kp(i) = wp(i)

  Ki(i) = max(wi(i), 0)

7. Store e(i), U(i), and w(i) for the next sample.

  Return Kp(i), Ki(i)

Fig 4 presents the flowchart of the proposed SMAPA-based adaptive PI controller, illustrating the sequence of the online adaptation and controller gain updating processes.

**Fig 4:**
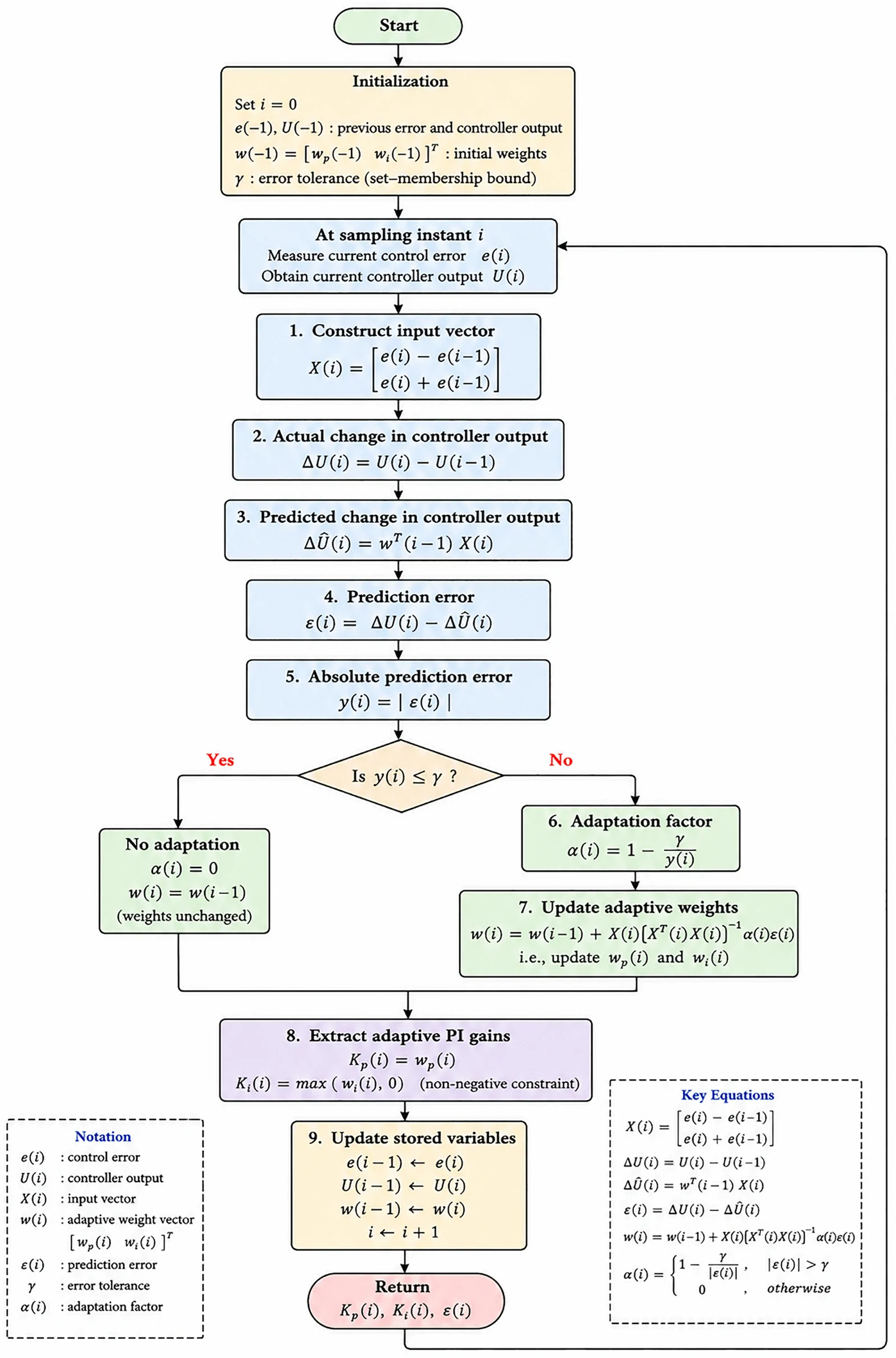
Flow chart of SMAPA controller.

### 4.3. Cascaded SMAPA – PID

The requirement to improve resilience and dynamic performance under changing operating conditions is what drives the adoption of a cascaded adaptive controller along with a traditional PID control technique in Load Frequency Control (LFC) systems. Conventional PID controllers, although simple and effective, are designed based on fixed system parameters and may exhibit degraded behavior during load disturbance conditions, system nonlinearities, and parameter variations inherent in power systems by incorporating an adaptive controller, such as the (SMAPA), within a cascaded structure, the control system can adjust its parameters online in response to changes in system dynamics and load variations. The conventional PID controller provides a fast response and ensures baseline system stability, while the adaptive component dynamically compensates for parameter uncertainties and external disturbances. This cascaded architecture leads to improved transient performance, reduced frequency overshoot, shorter settling time, and enhanced robustness compared to a standalone conventional PID controller. Consequently, the cascaded SMAPA–PID control strategy offers a more consistent and flexible recommendation for keeping frequency stability in new power systems.

## 5. Case studies

The coefficients of the cascaded SMAPA–PID controller are tuned using a structured one-factor-at-a-time procedure under the considered operating conditions. In this procedure, each controller coefficient is varied while the remaining coefficients are kept fixed, and its effect on the control performance is evaluated based on the IAE and transient response characteristics. This procedure is repeated sequentially for the different controller coefficients until a suitable set of parameters providing the best overall control performance is obtained. The selected coefficients are then kept fixed throughout the subsequent simulation and validation cases to ensure a consistent and reproducible evaluation.

The effectiveness and robustness of the proposed controller are investigated through a series of case studies, including a sudden load disturbance, random load variations, and wind-power disturbances. The proposed controller is evaluated in both single-area and two-area LFC configurations. In addition, a parameter uncertainty test is performed to assess the robustness of the controller against variations in the system parameters, while frequency-domain analysis is conducted to further examine its stability characteristics. The proposed controller is also compared with the recently reported SPPI-based approach under consistent operating conditions. For the two-area system, an additional real-time validation test is conducted to verify the practical feasibility of the proposed control strategy. In all cases, the obtained results are systematically compared with those of conventional controllers whose parameters are tuned using different optimization strategies.

### 5.1. Step change in load disturbance

As illustrated in Fig 5, the efficiency of the suggested controller for the single-area system is evaluated by subjecting one per unit sudden disturbance at ten seconds. A PI controller constructed with five optimizations—the genetic algorithm (GA), gravitational search algorithm (GSA), crow search algorithm (CSA), hybrid crow–GA and Crow–GSA approaches, and harmony search algorithm (HSA)—is used to compare the performance [29] as shown in Fig 6.

**Fig 5:**
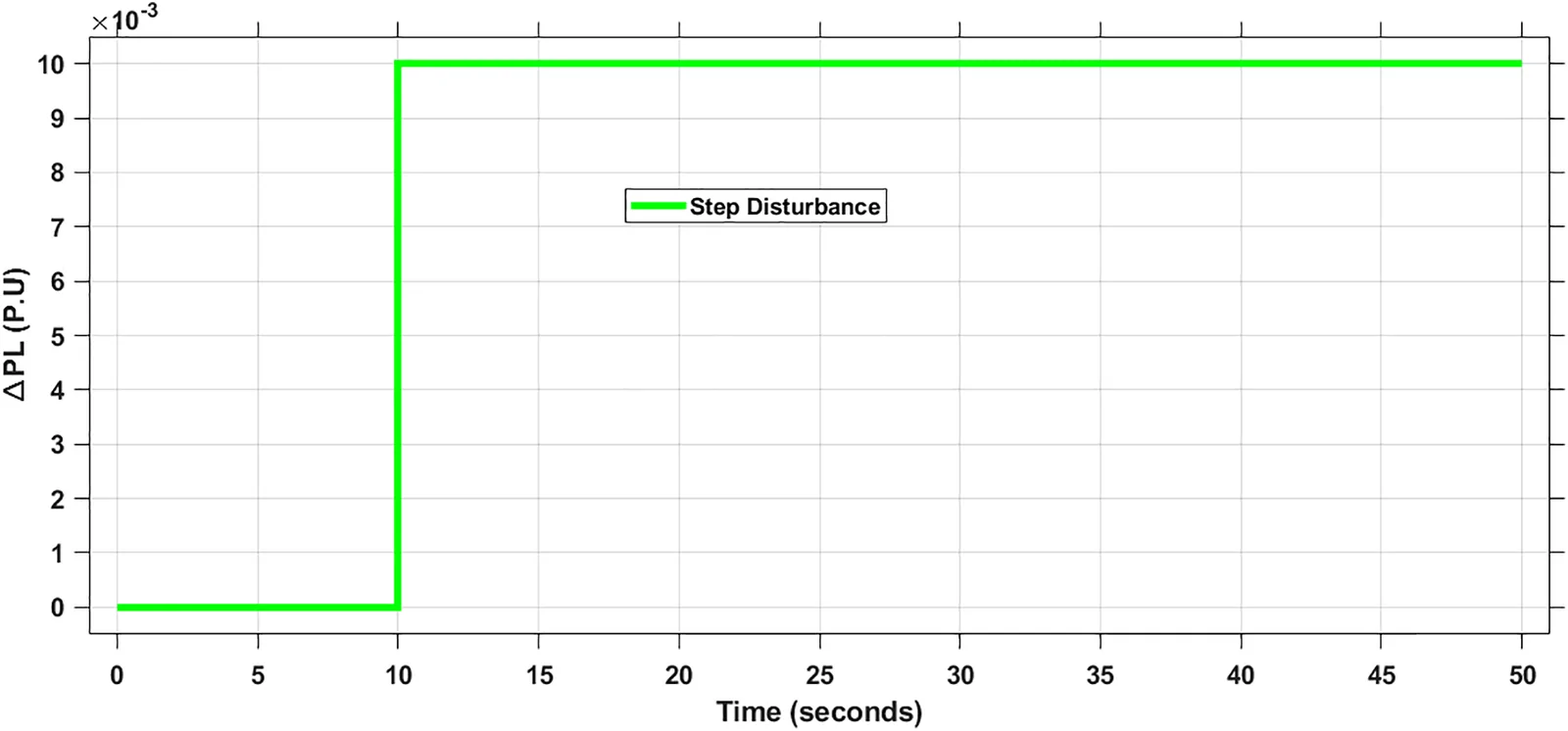
Case study 1 step disturbance.

**Fig 6:**
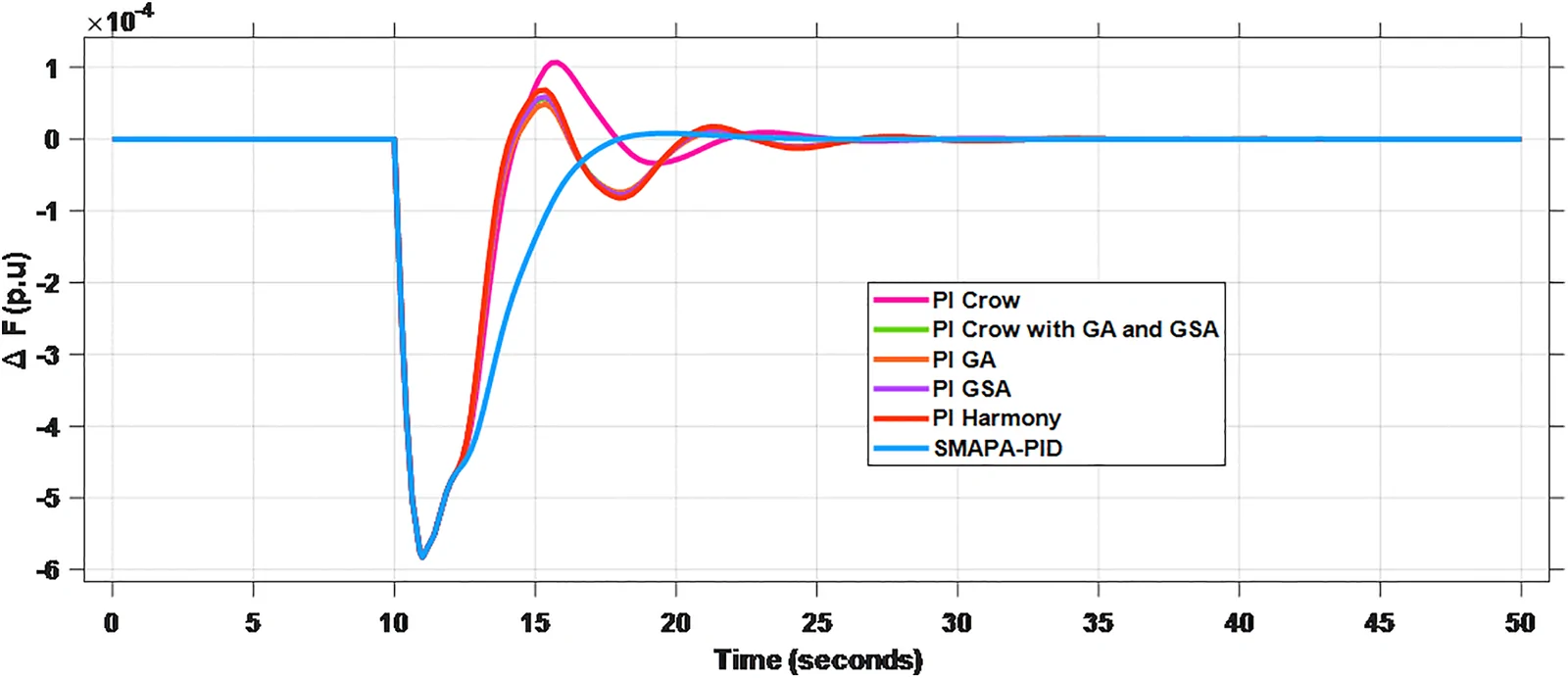
Case study 1 frequency change.

According to the findings, the introduced controller shows improved transient response and system stability relative to the other controllers since it has the lowest overshoot and settling time. Table 2 provides an overview of the controllers’ transient performance.

**Table 2:** Case study 1 Dynamic performance characteristics.

	SMAPA-PID	GA [29]	GSA [29]	CROW [29]	CROW-GA-GSA [29]	HARMONY [29]
**Over-shoot (OS)**	**5.496e-06**	**4.7e-05**	**5.78e-05**	**1.065e-04**	**5.544e-05**	**7.764 e-05**
**Under-shoot (US)**	**−5.82e-04**	**−5.82e-04**	**−5.82e-04**	**−5.82e-04**	**−5.82e-04**	**−5.82e-04**
**Settling time** $\tau_{s}$ **(sec)**	**12**	**16**	**16**	**16**	**16**	**16**
**IAE**	**0.00145**	**0.00160**	**0.00161**	**0.00166**	**0.00160**	**0.00163**

### 5.2. Random load variations

As seen in Figs 7 and 8, the effectiveness of the introduced controller is assessed through applying two random variation topologies of the load with practical operating conditions. The results are subsequently benchmarked against with PI controllers calibrated using five different algorithmic approaches, GA, GSA, Crow Search, hybrid Crow–GA/GSA, and Harmony Search [29], as illustrated in Figs 9 and 10.

**Fig 7:**
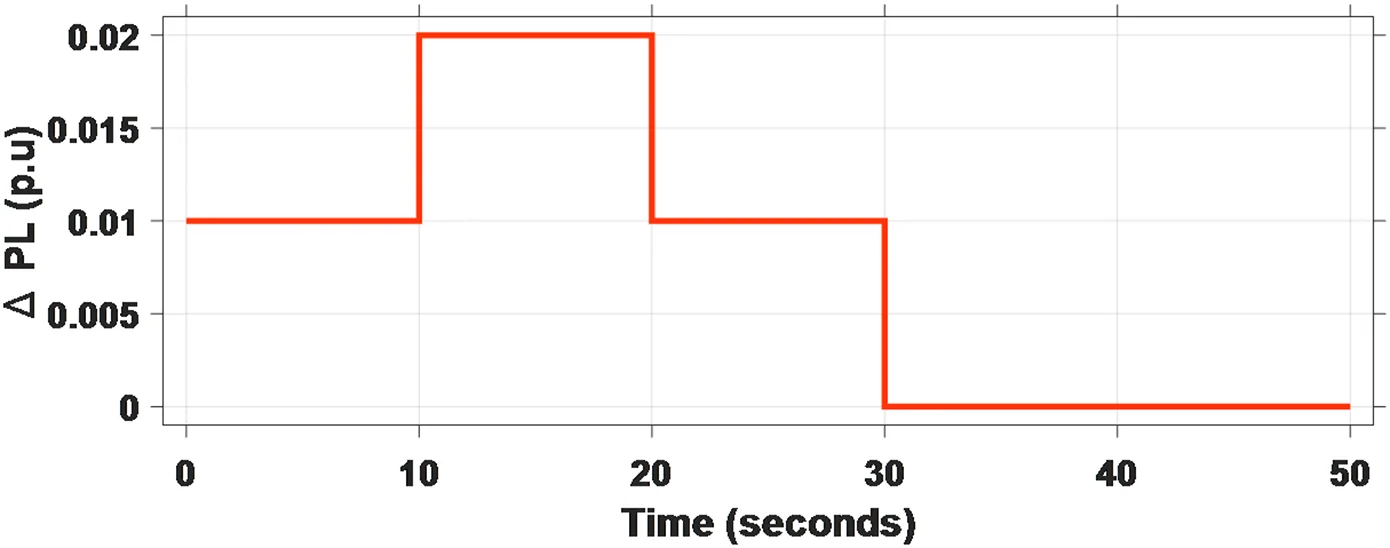
First random load variation.

**Fig 8:**
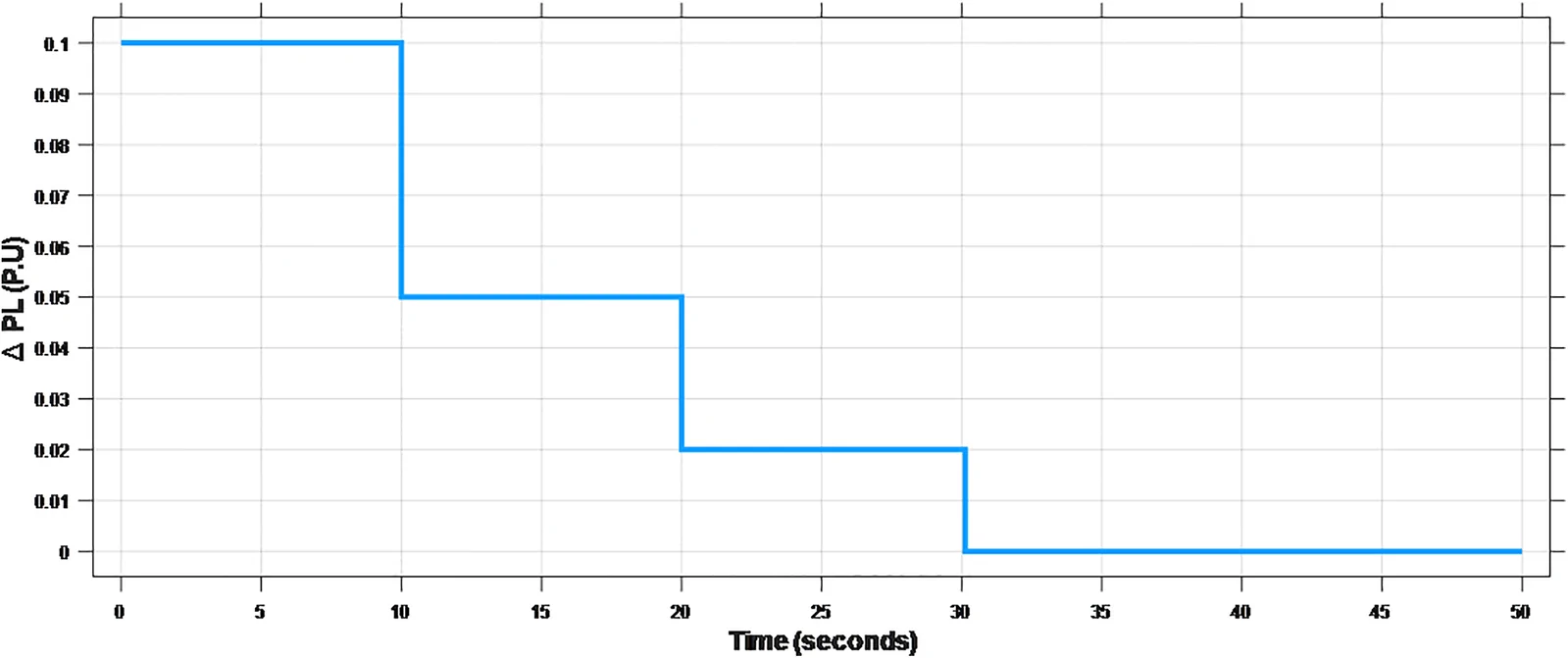
Second random load variation.

**Fig 9:**
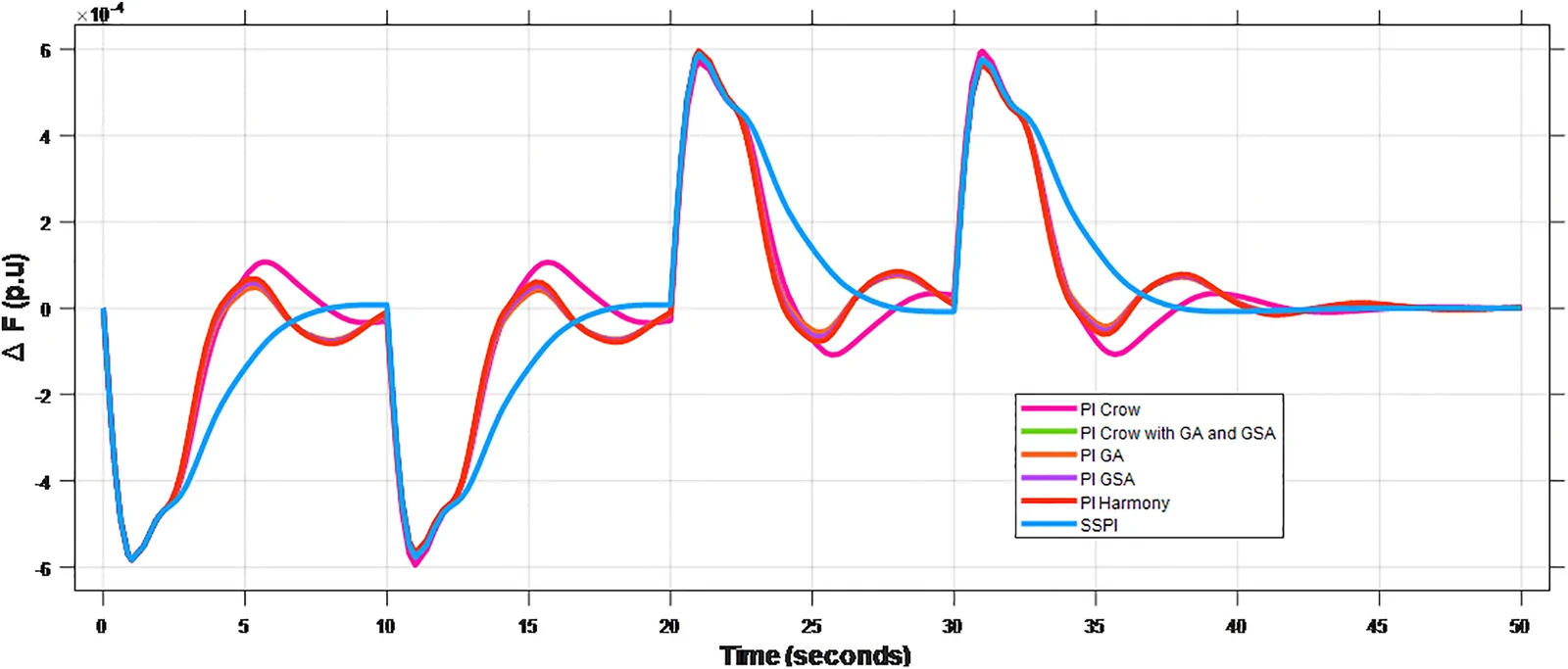
Case study 2 frequency change due to first random load.

**Fig 10:**
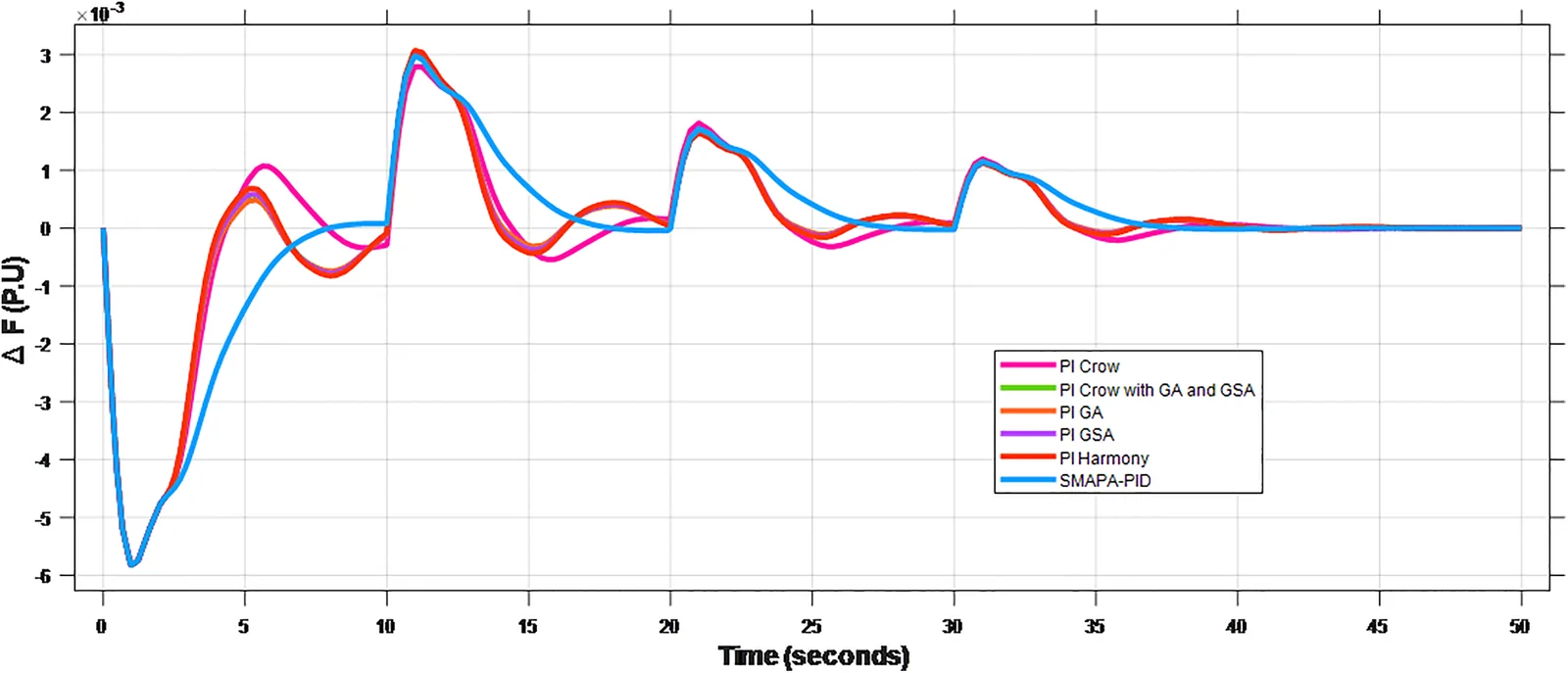
Case study 2 frequency change due to second random load.

The results of two load changes showed that the proposed controller exhibits little or comparable improvement in peak-to-peak overshoot; however, it achieves a significant improvement in settling time, which indicates superior transient performance and faster system stabilization. Tables 3 and 4 provide an overview of the transient performance of the controllers under consideration.

**Table 3:** Case study 2 dynamic performance characteristics under first random load.

	SMAPA-PID	GA [29]	GSA [29]	CROW [29]	CROW-GA-GSA [29]	HARMONY [29]
**Peak to peak Magnitude** $M_{pp}$	**1.16e-03**	**1.17e-03**	**1.173e-03**	**1.16e-03**	**1.172e-03**	**1.176e-03**
**Settling time** $\tau_{s}$ **(sec)**	**7.5**	**16**	**16**	**14.5**	**16**	**16**
**IAE**	**0.00422**	**0.00453**	**0.00455**	**0.00473**	**0.00454**	**0.00458**

**Table 4:** Second random load dynamic performance characteristics under second random load.

	SMAPA-PID	GA [29]	GSA [29]	CROW [29]	CROW-GA-GSA [29]	HARMONY [29]
**Peak to peak Magnitude** $M_{pp}$	**8.7e-03**	**8.82e-03**	**8.854e-03**	**8.7e-03**	**8.84e-03**	**8.89e-03**
**Settling time** $\tau_{s}$ **(sec)**	**8**	**14.5**	**14.5**	**13**	**14.5**	**14.5**
**IAE**	**0.0247**	**0.0267**	**0.0269**	**0.0277**	**0.0268**	**0.0277**

### 5.3. Wind speed variation

To examine the performance characteristics of the introduced controller in interconnected systems, a wind speed deviation illustrated in Fig 11 is subjected to the system, resulting in a corresponding wind turbine disturbance as shown in Fig 12.The results are then benchmarked against a PI controller adjusted with five techniques: GA, GSA, hybrid Crow-GA–GSA, and Harmony [29], as illustrated in Fig 13.

**Fig 11:**
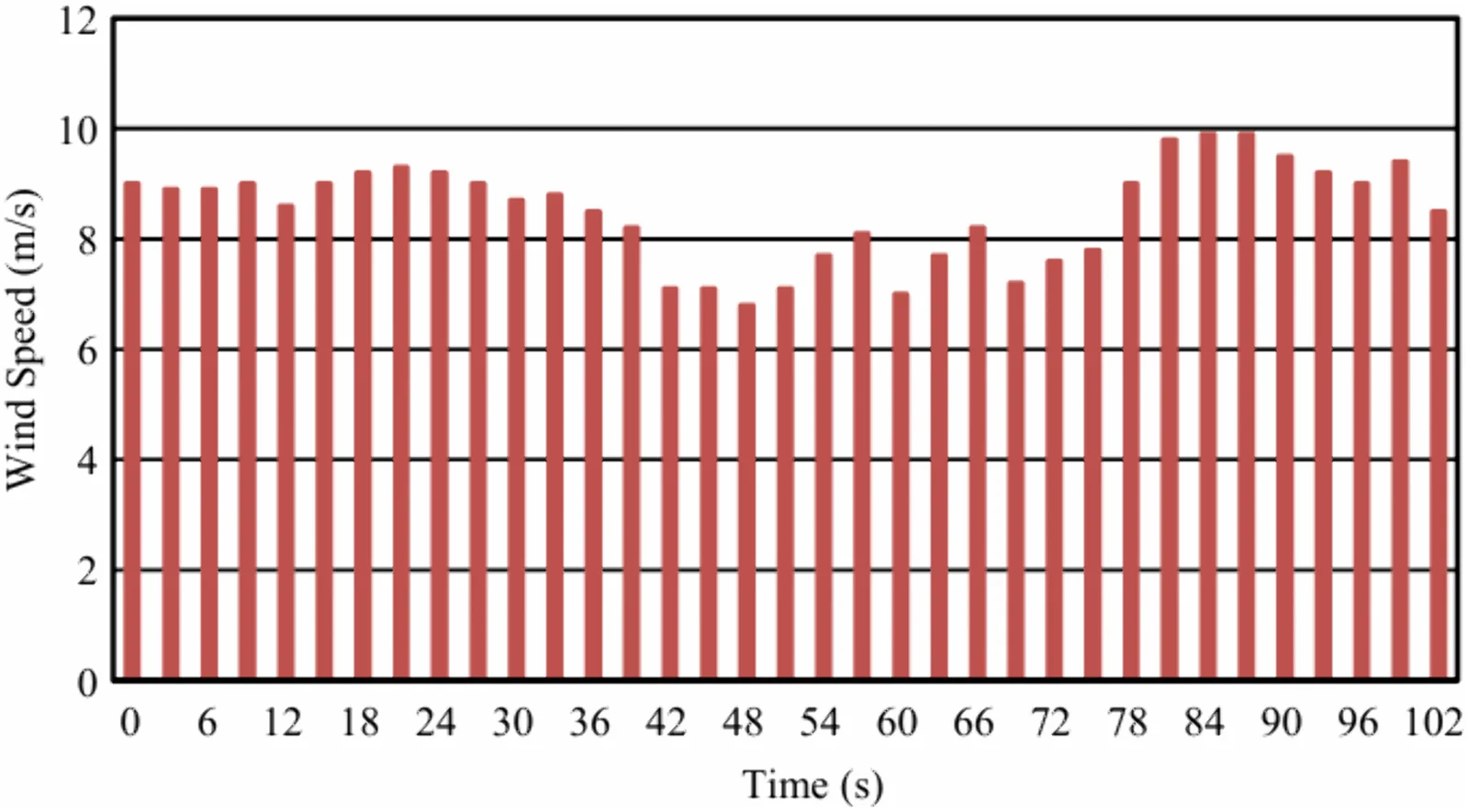
Wind speed fluctuation.

**Fig 12:**
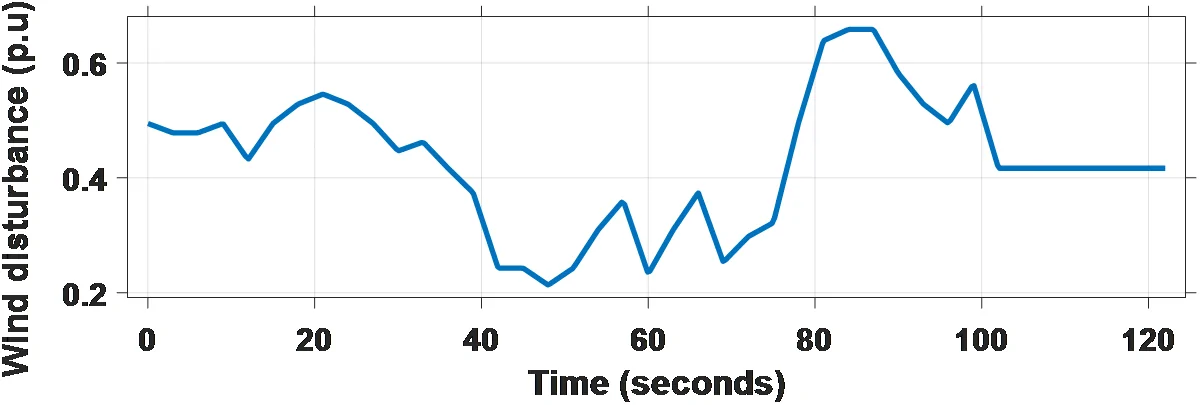
Wind generator disturbance.

**Fig 13:**
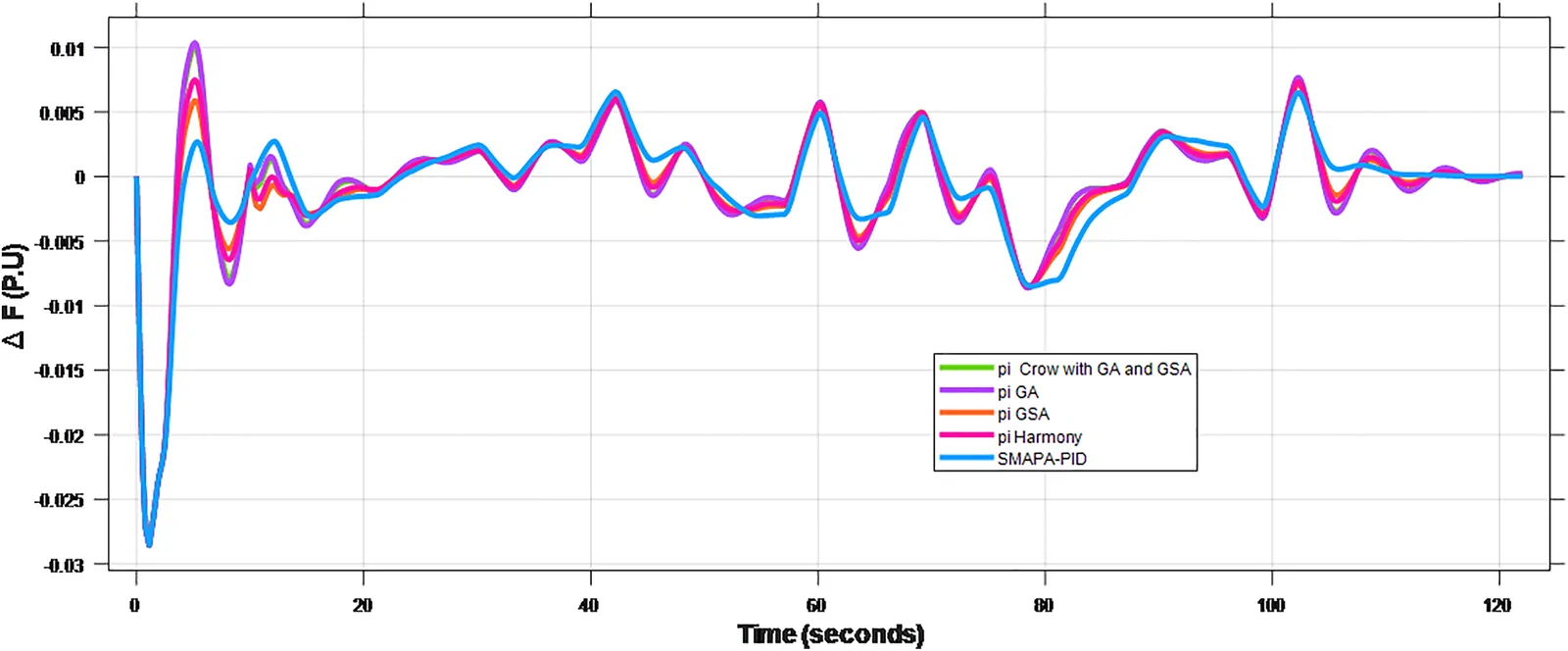
Case study 3 frequency change.

The output graphs confirmed that the controller under study accomplish reduced magnitude of peak-to-peak and reduced steady state time relative to the other controllers with renewable energy disturbances, which indicates the robustness of the controller with renewable resource conditions. A summary of the transient behavior of the controllers considered is presented in Table 5.

**Table 5:** Case Study 3 dynamic performance characteristics.

	SMAPA-PID	GA [29]	GSA [29]	CROW-GA-GSA [29]	HARMONY [29]
**Peak to peak Magnitude** $M_{pp}$	**3.3e-02**	**3.847e-02**	**3.416e-02**	**3.815e-02**	**3.571e-02**
**Settling time** $\tau_{s}$ **(sec)**	**10**	**17**	**17**	**17**	**17**
**IAE**	**0.1964**	**0.2031**	**0.1972**	**0.2012**	**0.1972**

### 5.4. Step load change in a two-area power system

As illustrated in Fig 5 for the two-area system, the controller parameters are tuned using a trial and error approach with one per unit sudden load change occurred at ten seconds at Area 1 to evaluate the steadiness of the studied cascaded controller in a interconnected system. The achieved simulation outputs are subsequently benchmarked with those obtained using a traditional PID controller adjusted by the TLBO, HS, and SCA algorithms [24], as presented in Figs 14 and 15.

**Fig 14:**
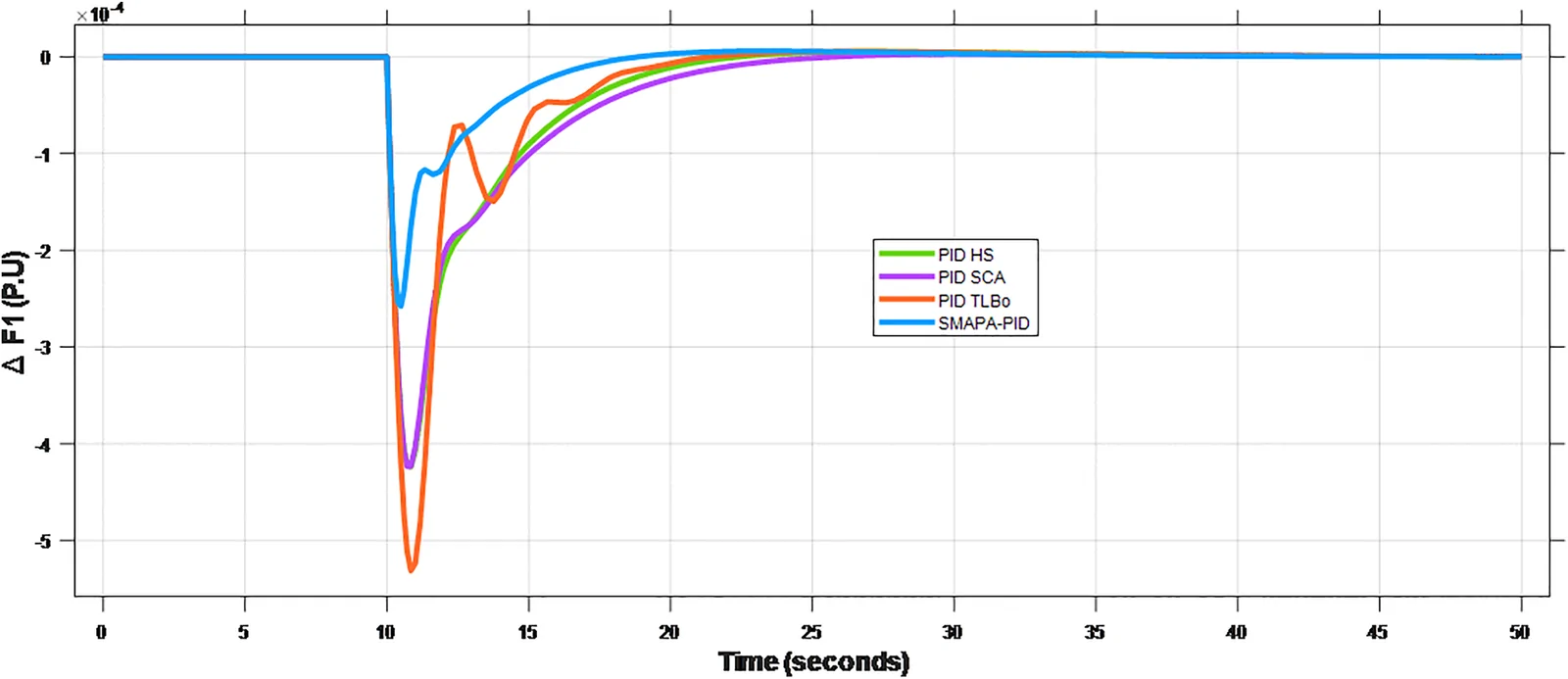
Case study 4 frequency change at Area 1.

**Fig 15:**
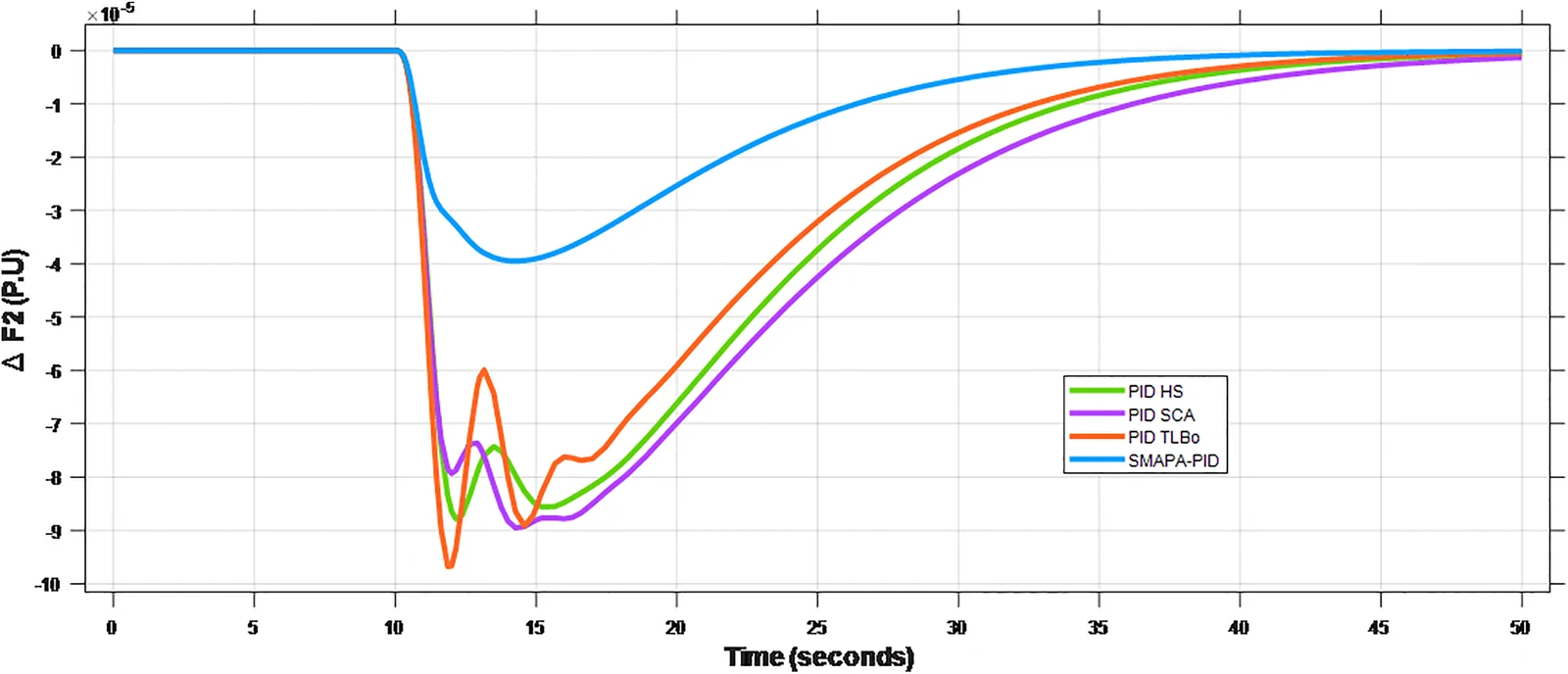
Case study 4 frequency change at Area 2.

The results indicate that the introduced controller significantly enhances dynamic behavior relative to the other controllers by achieving reduced overshoot and faster settling of the frequency error response in both areas under step load disturbance conditions. The dynamic response characteristics for Case 4 are presented in Table 6. A comparison of the IAE performance indices for all controllers is presented in Table 7.

**Table 6:** Case study 4 dynamic performance characteristics.

	Area one				Area Two			
	SMAPA-PID	TLBO [22]	SCA [22]	HS [22]	SMAPA-PID	TLBO [22]	SCA [22]	HS [22]
**Under-shoot (US)**	**−2.54e-04**	**−5.30e-04**	**−4.23e-04**	**−4.22e-04**	**−3.94e-05**	**−9.64e-05**	**−8.95e-05**	**−8.76e-05**
**Settling time** $\tau_{s}$ **(sec)**	**10**	**15**	**15**	**15**	**32.5**	**37**	**37**	**37**

**Table 7:** Performance index comparison.

controller	IAE
SSPI	0.00255
PID TLBO	0.00537
PID SCA	0.00608
PID HS	0.00575

### 5.5. Two area system random load variations

The purpose of these case studies is to test how well the suggested controller performs in an interconnected system (a two-area LFC system) in comparison to the single-area system. Two different irregular load changes, representing practical operating case in real power systems, are subjected to Area 1, as displayed in Figs 7 and 9. As illustrated in Figs 16–19, the functionality of the studied cascaded controller is contrasted with that of traditional PID controllers adapted using the TLBO, SCA, and HS algorithms [22**]**.

**Fig 16:**
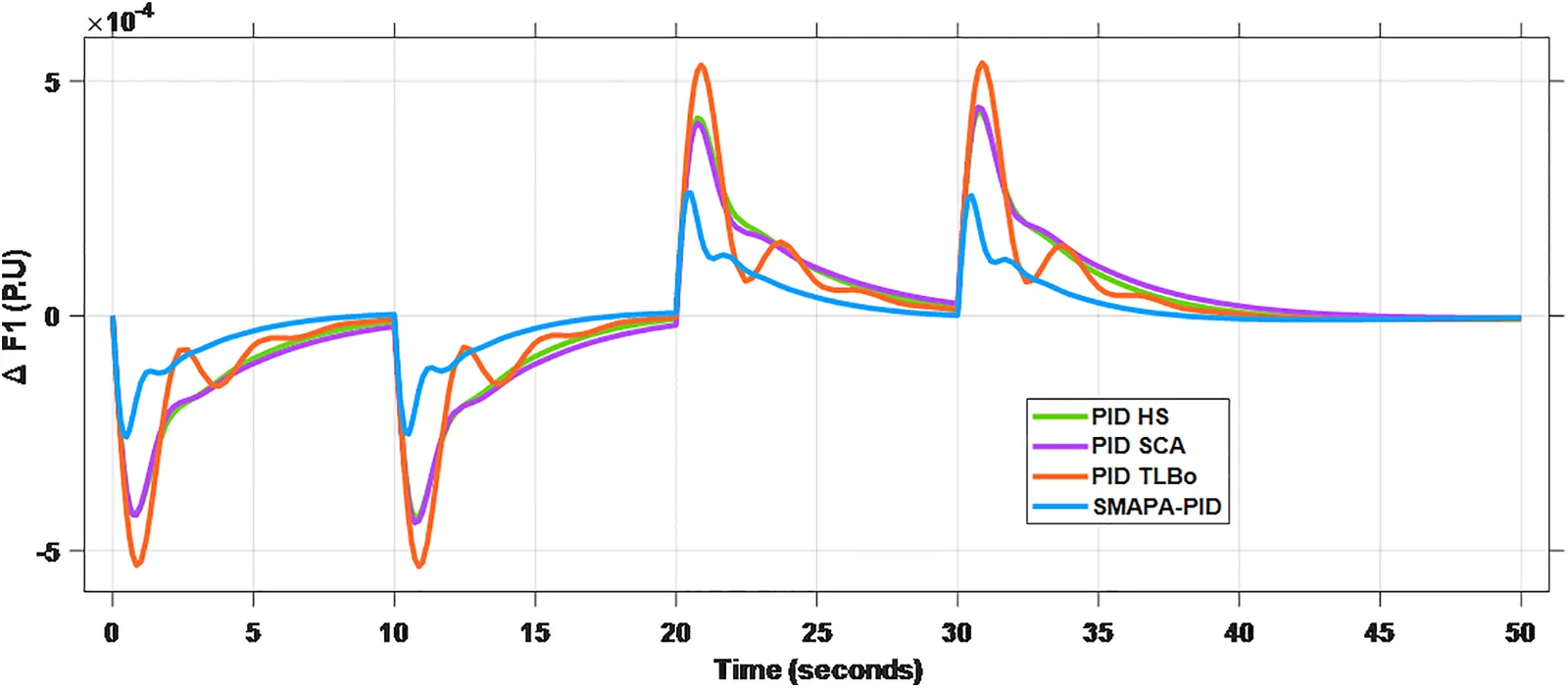
Case study 5 frequency change at Area 1 due to the first random load.

**Fig 17:**
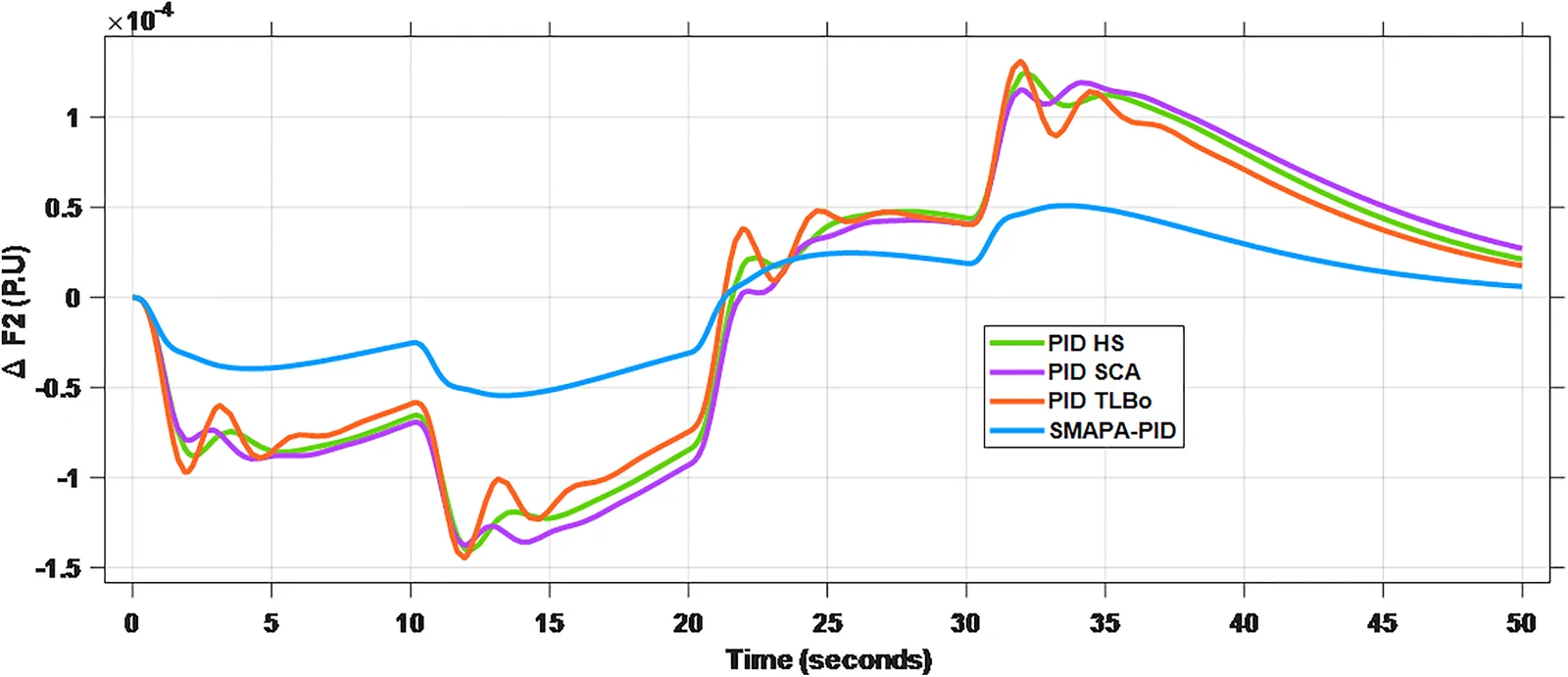
Case study 5 frequency change at Area 2 due to the first random load.

**Fig 18:**
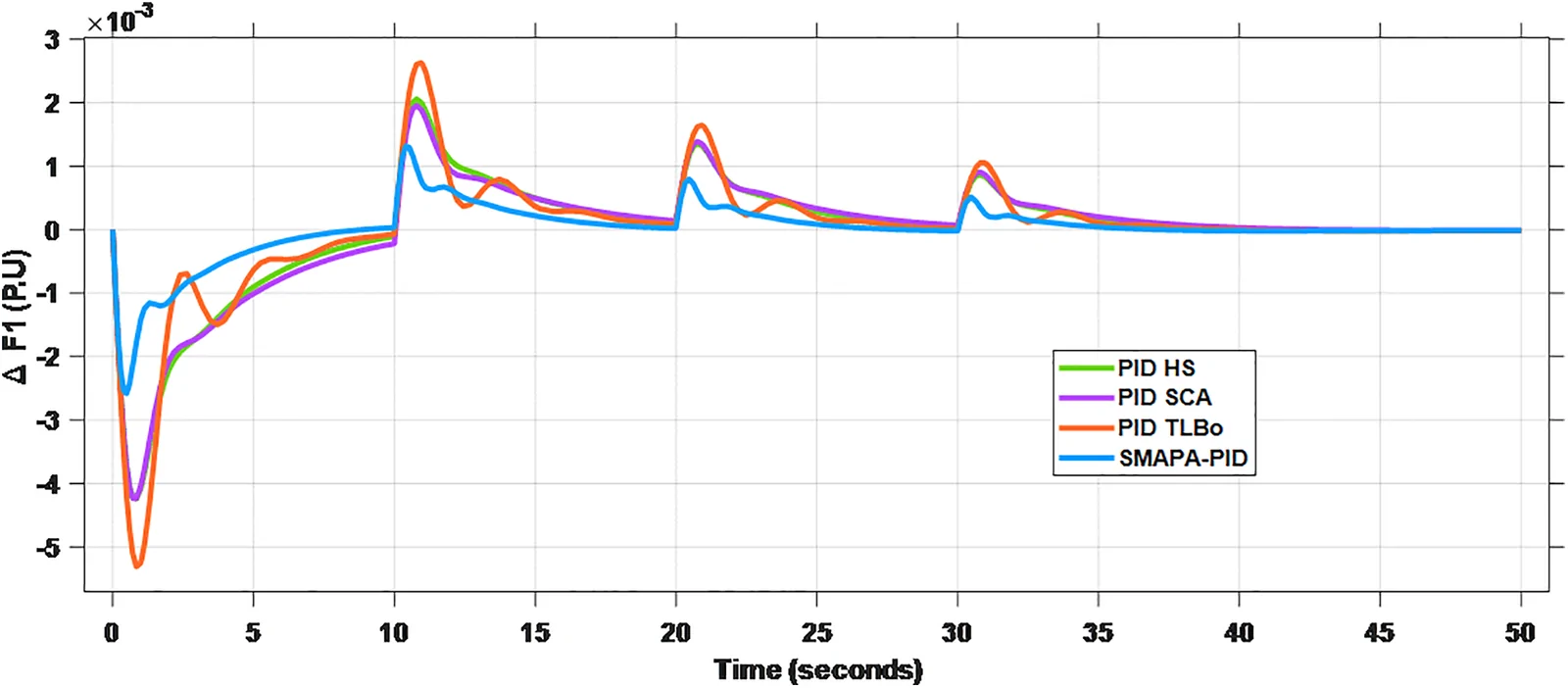
Case study 5 frequency change at Area 1 due to the second random load.

**Fig 19:**
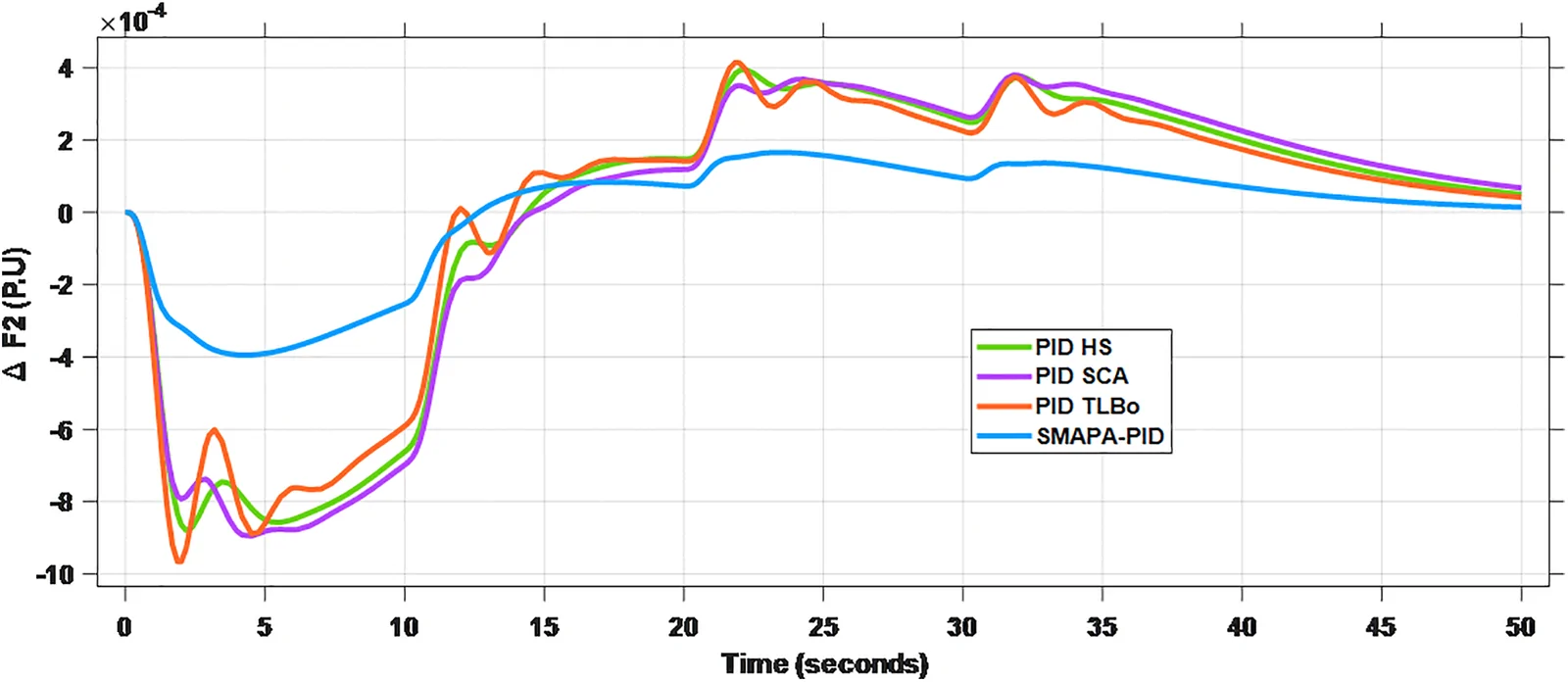
Case study 5 frequency change at Area 2 due to the seond random load.

The results demonstrate that the overshoot and settling time of frequency variation signals are effectively reduced by action of the developed controller, thereby improving the overall dynamic performance under two random load variations, which represent practical scenarios in real power systems. The transient responses for the two cases are illustrated in Tables 8 and 9. A comparison of the IAE performance indices for all controllers is presented in Tables 10 and 11.

**Table 10:** Performance index comparison.

controller	IAE
SSPI	0.01586
PID TLBO	0.0327
PID SCA	0.0355
PID HS	0.0347

**Table 11:** Performance index comparison.

controller	IAE
SSPI	0.0829
PID TLBO	0.1684
PID SCA	0.1800
PID HS	0.1785

**Table 8:** Case study 5 dynamic performance characteristics under the first random load.

	Area one				Area Two			
	SMAPA-PID	TLBO [22]	SCA [22]	HS [22]	SMAPA-PID	TLBO [22]	SCA [22]	HS [22]
**Peak to peak Magnitude** $M_{pp}$	**5.16e-04**	**1.06e-03**	**8.31e-04**	**8.43e-04**	**1.05e-04**	**2.73e-04**	**2.55e-04**	**2.64e-04**
**Settling time** $\tau_{s}$ **(sec)**	**7.5**	**12**	**12.5**	**12.5**	**10.5**	**17**	**19**	**18**

**Table 9:** Case study 5 dynamic performance characteristics under the second random load.

	Area one				Area Two			
	SMAPA-PID	TLBO [22]	SCA [22]	HS [22]	SMAPA-PID	TLBO [22]	SCA [22]	HS [22]
**Peak to peak Magnitude** $M_{pp}$	**3.84e-03**	**7.91e-03**	**6.18e-03**	**6.29e-03**	**5.46e-04**	**1.38e-03**	**1.27e-03**	**1.27e-03**
**Settling time** $\tau_{s}$ **(sec)**	**5.5**	**7.5**	**10**	**10**	**10**	**16.5**	**20**	**18**

### 5.6. Area system wind speed variation

To analyze the behavior of the proposed controller under wind energy variations, which represents a practical scenario in modern power systems, wind power output fluctuations are implemented in area 1, as illustrated in Fig 11. A comparison is then carried out with PID controllers tuned via TLBO, SCA, and HS techniques [22], as shown in Figs 20–21. A comparison of the IAE performance indices for all controllers is presented in Table 12.

**Table 12:** Performance index comparison.

controller	IAE
SSPI	0.7425
PID TLBO	1.5539
PID SCA	1.7484
PID HS	1.6672

**Fig 20:**
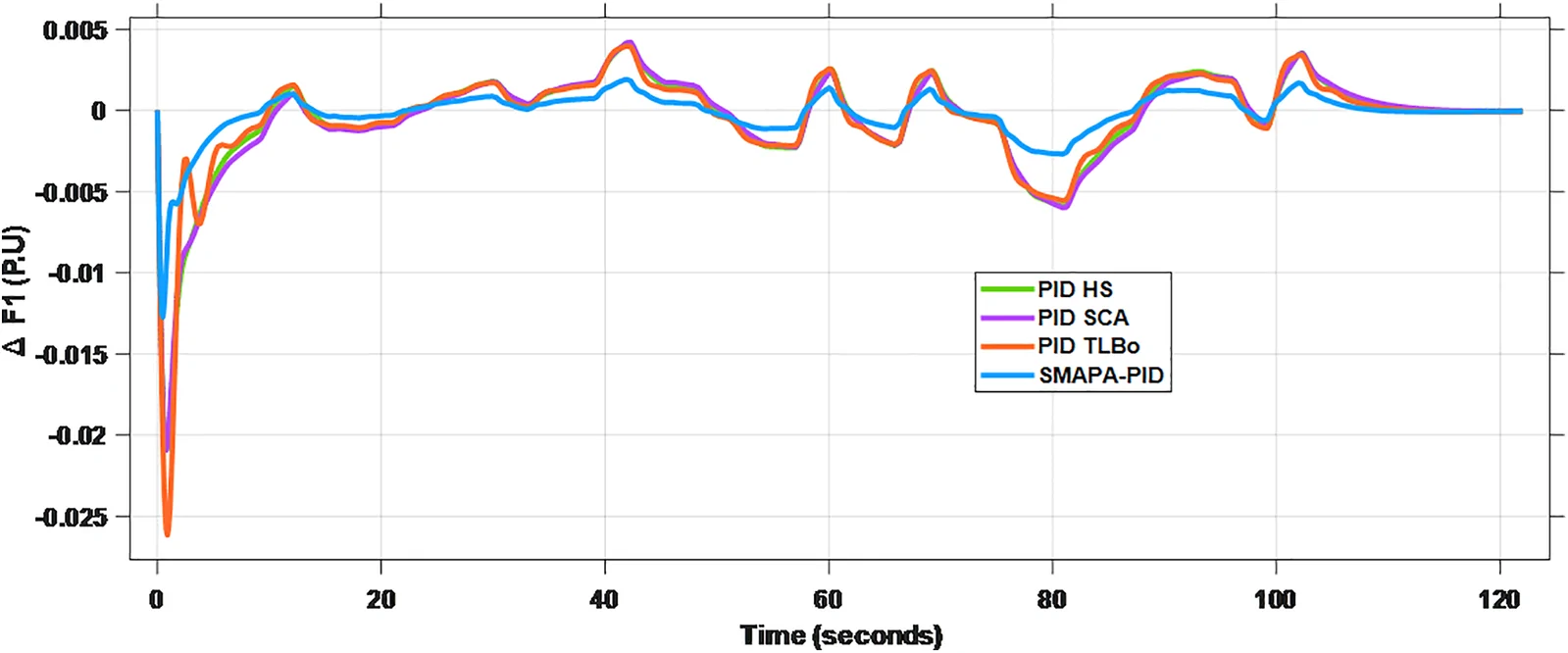
Case study 6 frequency change at Area 1.

**Fig 21:**
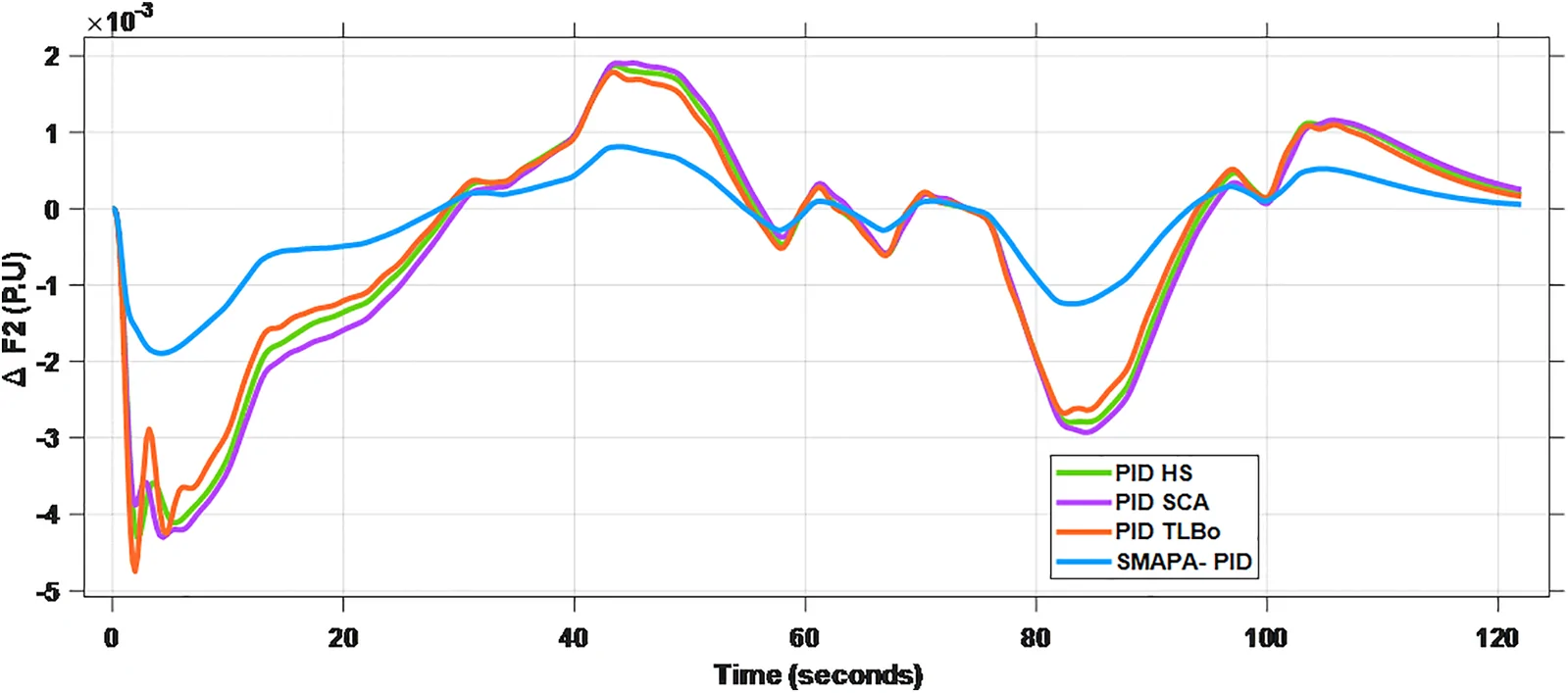
Case study 6 frequency change at Area 2.

The advantage of the proposed controller is demonstrated by the results, which show that it achieves smaller overshoot and shorter settling time under wind generating disturbances when compared to the other controllers examined in this study. Table 13 provides a summary of the dynamic performance.

**Table 13:** Case study 6 dynamic performance characteristics.

	Area one				Area Two			
	SMAPA-PID	TLBO [22]	SCA [22]	HS [22]	SMAPA-PID	TLBO [22]	SCA [22]	HS [22]
**Peak to Peak shoot**	**1.44e-02**	**3.01e-02**	**2.5e-02**	**2.5e-02**	**2.69e-03**	**6.52e-03**	**6.16e-03**	**6.17e-03**
**Settling time (sec)**	**9**	**10.5**	**12**	**12**	**12.5**	**19**	**20**	**21**

### 5.7. Real time validation test for two area system with step load disturbance

In this case study, the dynamic performance and stability of the proposed controller are validated through real-time experimentation using a real-time simulator. Case Study 4, corresponding to one per unit sudden load change occurred at ten seconds in Area one of the two-area power system, is implemented for real-time validation. The power system model is developed and executed in MATLAB/Simulink and interfaced with a Speedgoat real-time simulator. The experimental responses are acquired using a Fluke ScopeMeter, which is connected to the Speedgoat real-time simulator through an I/O interface module. The overall experimental setup is illustrated in Fig 22.

**Fig 22:**
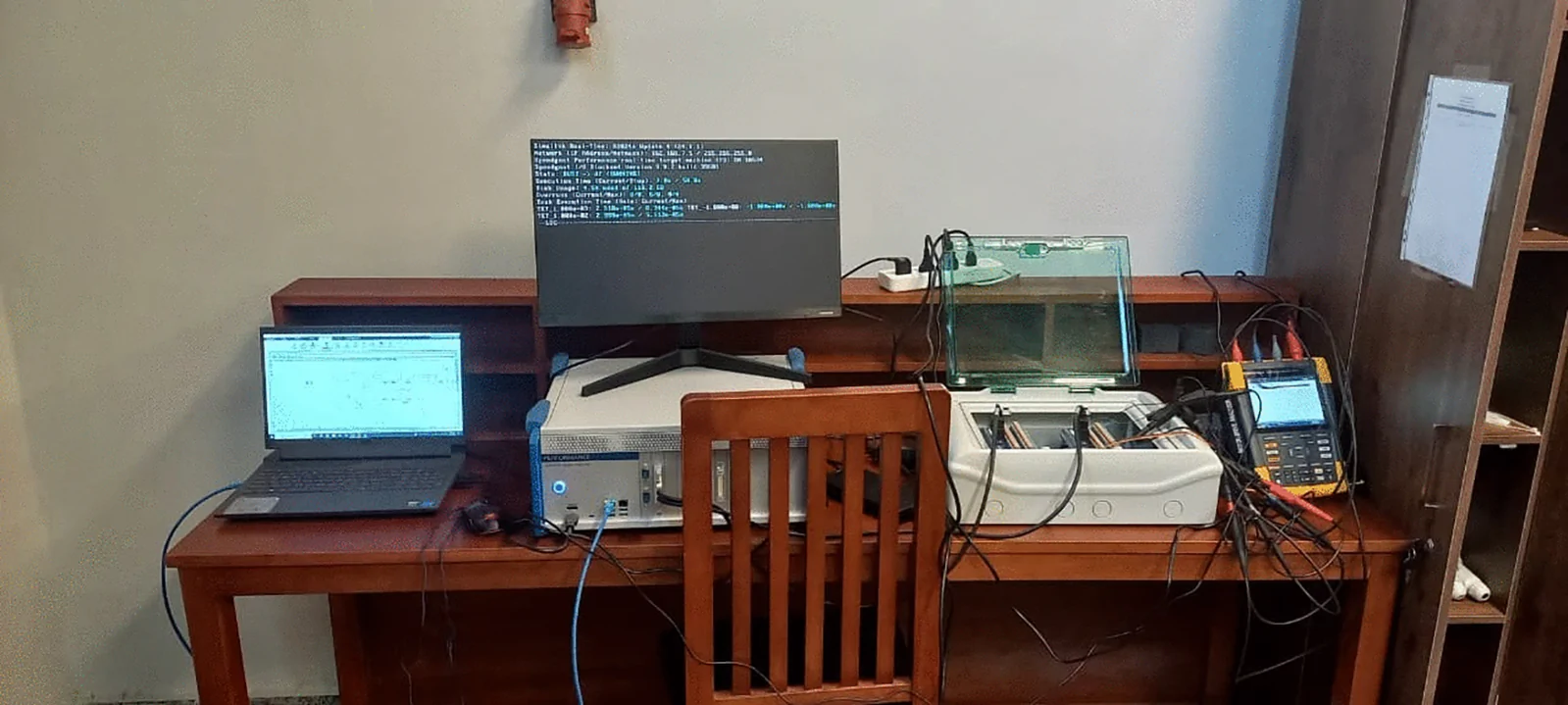
Experimental setup. Image from [Alharbi et al.], [2026], distributed under a Creative Commons Attribution (CC BY 4.0) license.

The real time results verified the simulation results and proved the controller robustness, stability and superior transient behavior measured against conventional PID controllers optimized using different optimization methods as shown in Figs 23 and 24.

**Fig 23:**
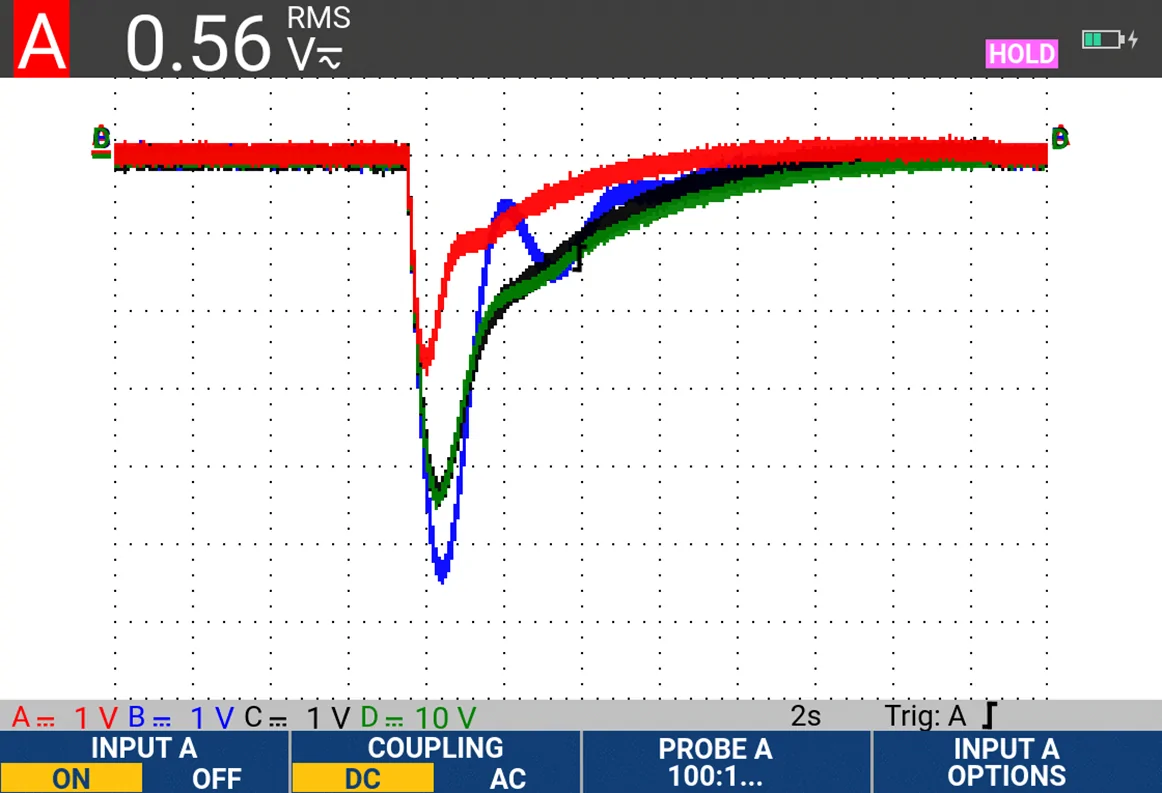
Frequency change in area 1.

**Fig 24:**
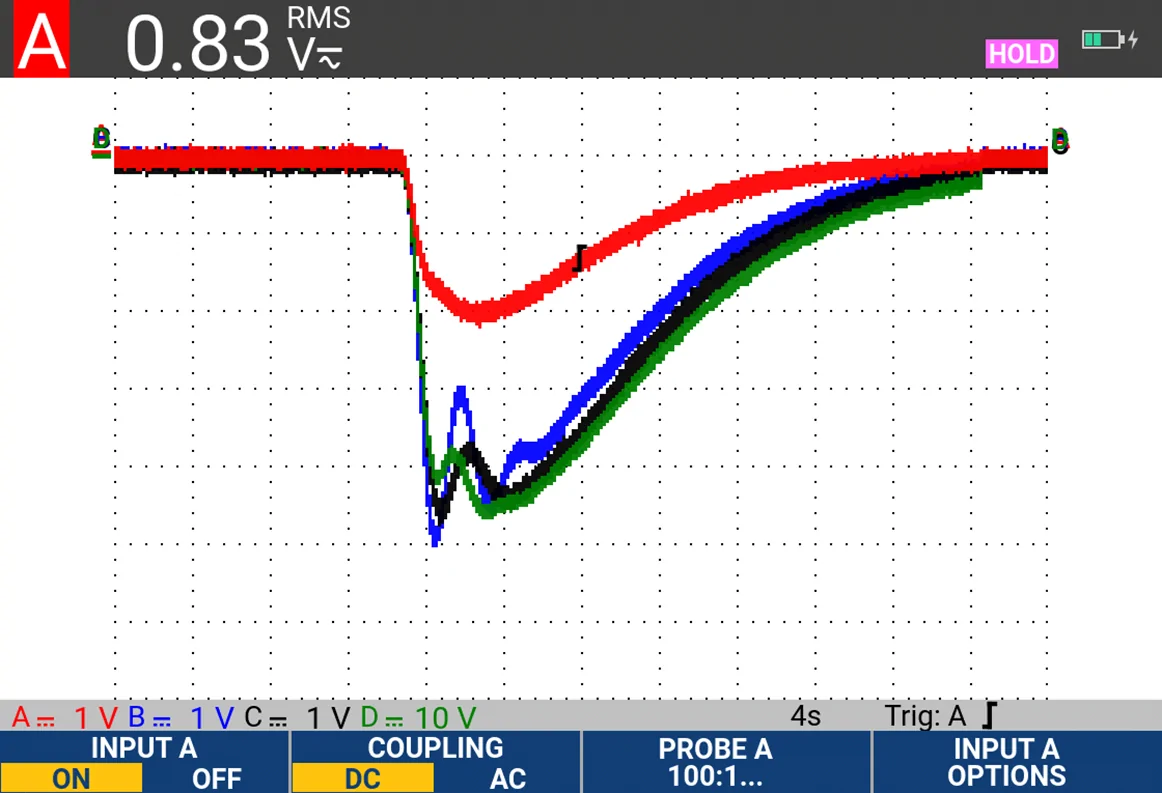
Frequency change in area 2.

To assess the computational requirements of the proposed controller and its suitability for real-time implementation, a comparative computational complexity analysis was conducted against the conventional PI and PID controllers. The comparison is summarized in Table 14.

**Table 14:** Controllers comparison.

Controller	Number of Tunable Parameters	Main Computational Operations	Computational Burden	Suitability for Real-Time Implementation
PI	2	Proportional and integral calculations	Low	High
PID	3	Proportional, integral, and derivative calculations	Low	High
Proposed SMAPA–PID	2 adaptive parameters + 3 PID parameters	Online adaptive weight update, error processing, gain generation, and conventional PID calculations	Moderate	High

The proposed SMAPA–PID controller involves additional computations due to the online adaptive mechanism. Nevertheless, its computational structure remains suitable for real-time implementation, particularly since the adaptive update involves a limited number of parameters.

### 5.8. Comparison with modern adaptive controller

To further evaluate the proposed controller against a recent state-of-the-art adaptive LFC strategy, a comparative case study with the recently published SPPI-based controller is conducted under random load variations as Fig 6 [20]. Both controllers are evaluated under identical operating conditions using the same performance metrics. The corresponding comparison results are presented in the Figs 25 and 26, while the transient performance metrics of both controllers are summarized in Table 15.

**Table 15:** Dynamic performance summary.

	SMAPA-PID		SSPI-PID	
	∆F1	∆F2	∆F1	∆F2
**Peak to peak Magnitude $M_{pp}$ *M_pp_***	**5.17e-04**	**5.95e-05**	**7.88e-04**	**1.48e-04**
**Settling time $\tau_{s}$ (sec)**	**3.55**	**25**	**3.55**	**30**
**IAE**	**0.0078**		**0.0172**	

The results show that the proposed controller achieves better performance than the recently published SPPI-based controller in terms of peak-to-peak shoot, settling time and IAE.

**Fig 25:**
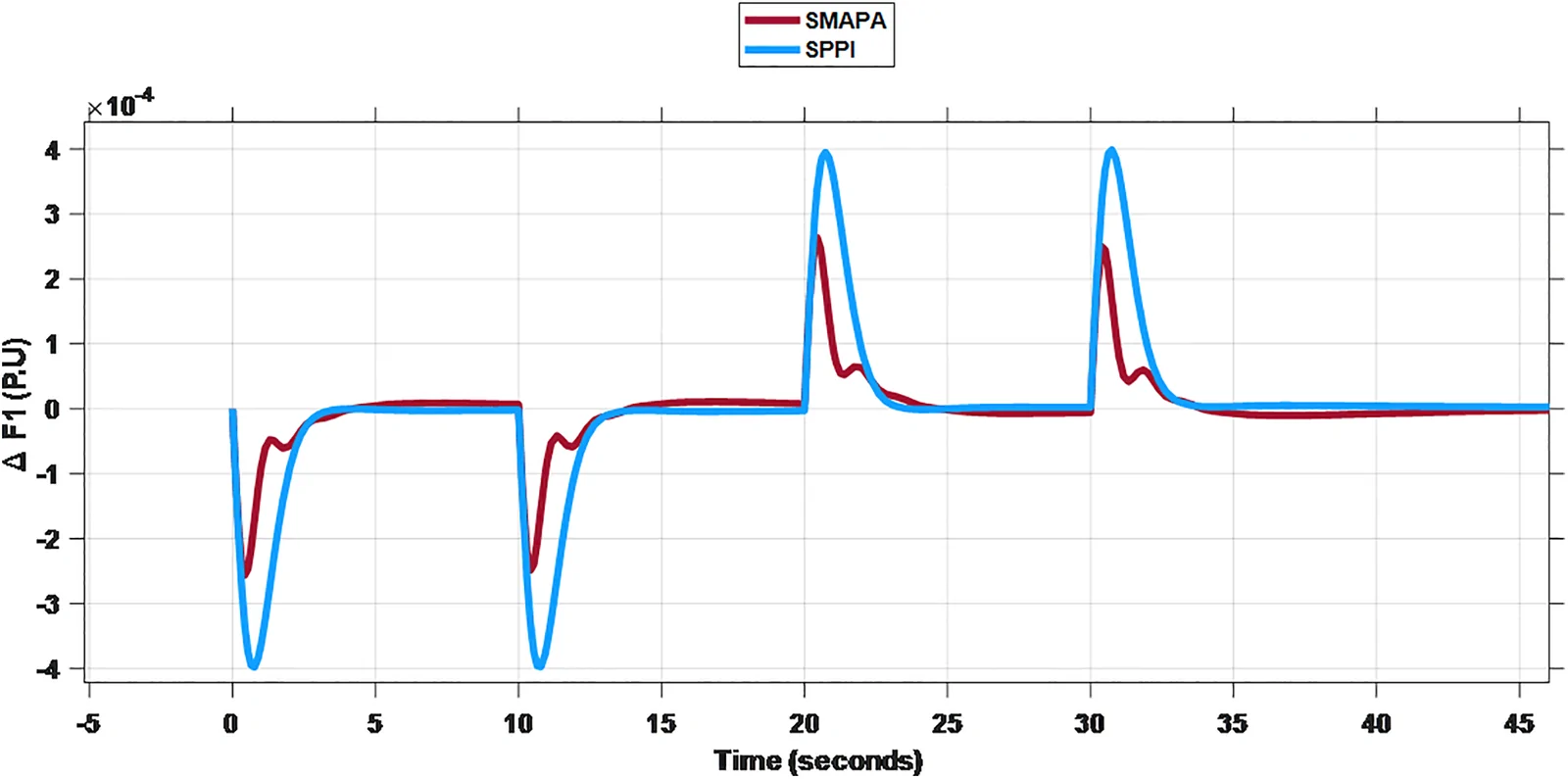
Case study 8 frequency change in area 1.

**Fig 26:**
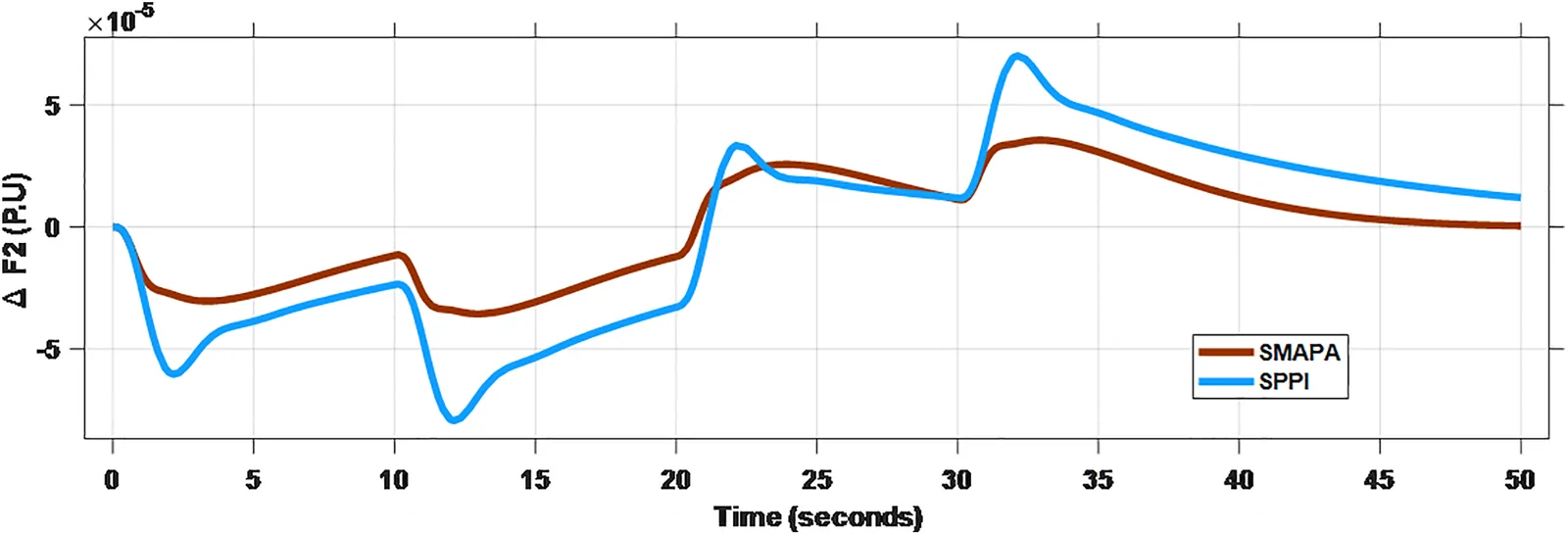
Case study 8 frequency change in area 2.

### 5.9. Parameter uncertainty test

To evaluate the robustness of the proposed SMAPA–PID controller against variations in the power-system parameters, a parameter uncertainty analysis is conducted. In this case, the nominal values of the speed regulation coefficient, generator inertia constant, and load damping coefficient are independently varied within a range of their nominal values. The controller parameters are kept unchanged during the uncertainty analysis, while the system parameters are varied from their nominal values. This test is performed to examine whether the proposed controller can maintain satisfactory frequency regulation performance when the actual system parameters deviate from their nominal values. The resulting frequency deviations and transient responses are presented in Fig 27 and 28, while the corresponding performance metrics are summarized in Table 16.

**Table 16:** Dynamic performance summary.

Settling Time (sec)	Peak to peak Overshoot (p.u)	Settling Time (sec)	Peak to peak Overshoot	Parameter Variation
Area 2		Area 1		
30	5.98e-05	3.55	5.15e-04	Nominal
32	6.16e-05	3.82	4.81e-04	M + 20%
31	5.74e-05	3.78	5.65e-04	M −20%
31	5.97e-05	3.78	5.14e-04	D + 20%
32	5.99e-05	3.88	5.17e-04	D – 20%
37	5.83e-05	3.92	5.1e-04	R + 20%
36	6.14e-05	3.64	5.22e-04	R −20%

The results show that the variations in the system parameters produce only minor changes in the transient response, which are barely noticeable compared with the nominal case. This indicates that the proposed controller maintains consistent performance despite the considered parameter uncertainties.

**Fig 27:**
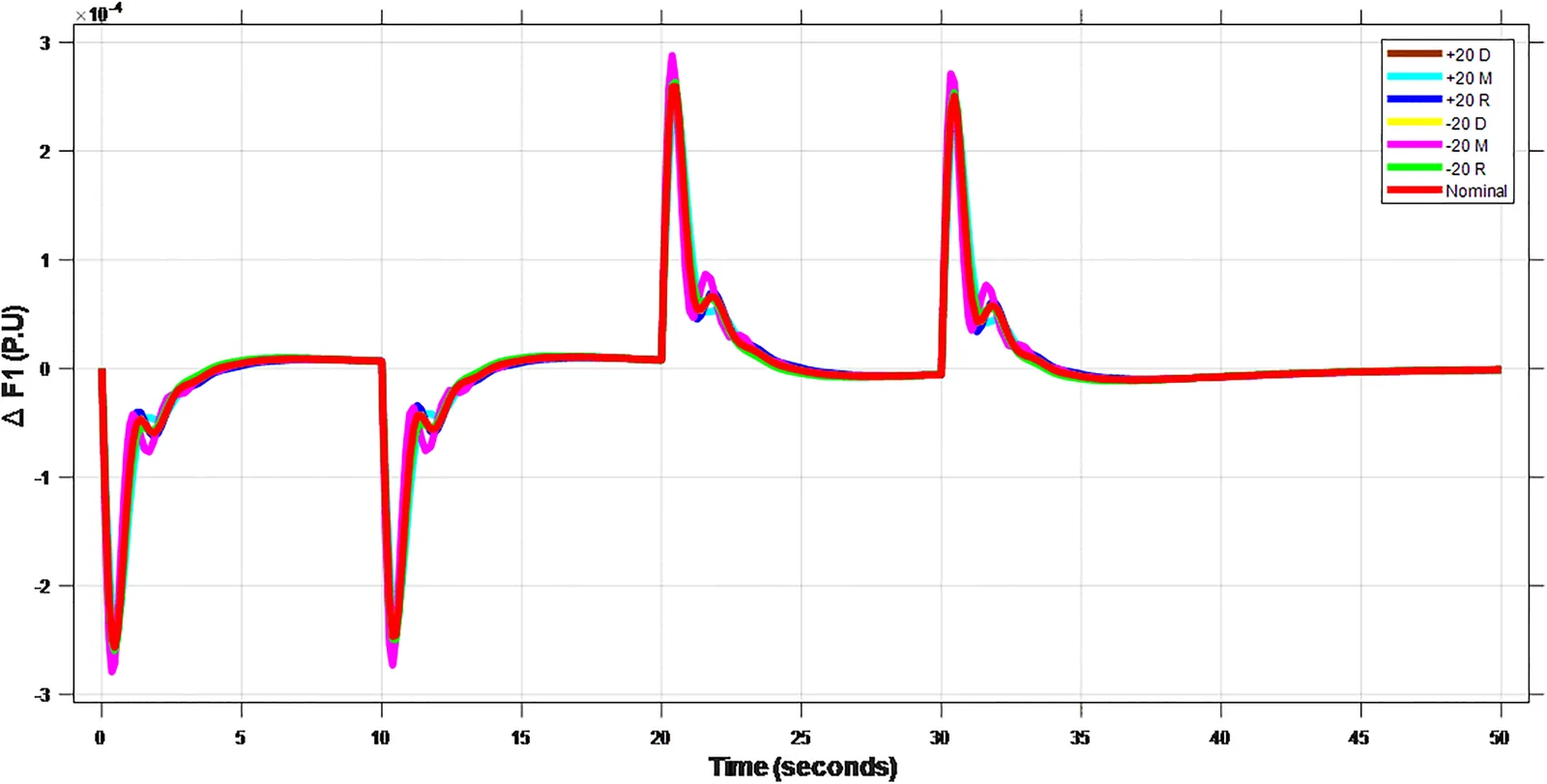
Case study 9 frequency change in area 1.

**Fig 28:**
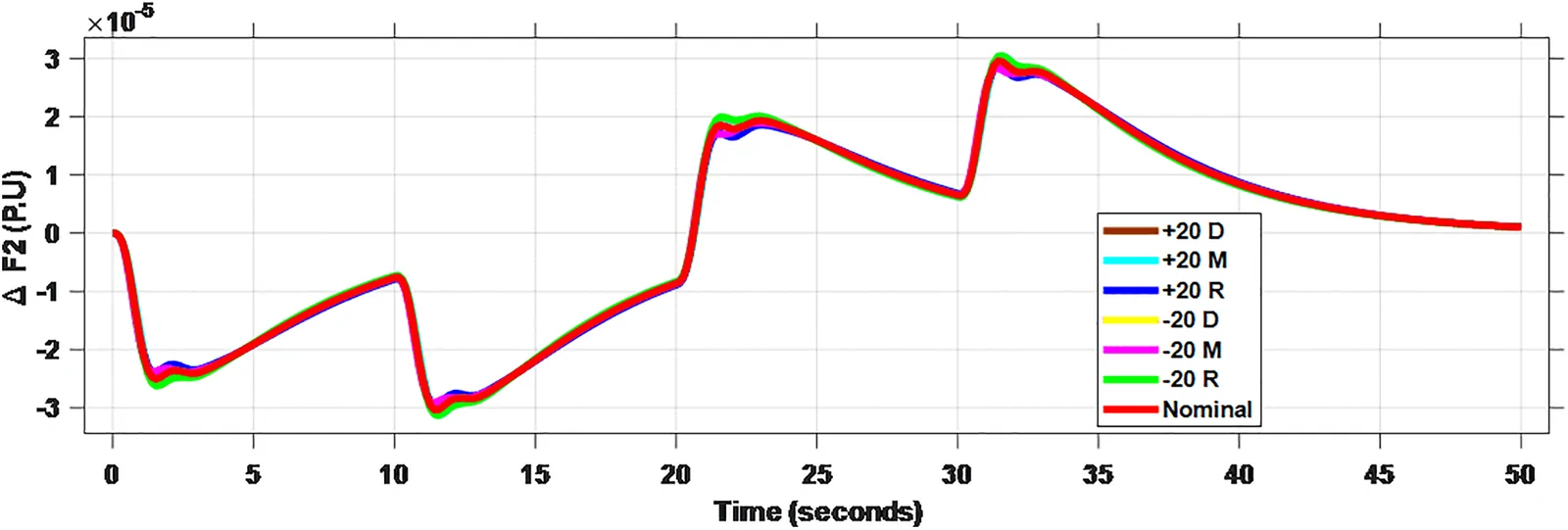
Case study 9 frequency change in area 2.

### 5.10. Frequency-domain stability analysis

To further assess the stability and robustness of the proposed cascaded SMAPA–PID controller, a frequency-domain analysis was performed using the Bode diagram. As shown in Fig 29, the controlled system exhibits a gain margin of 30.66 dB and a phase margin of 40. 57°.The positive gain and phase margins indicate that the considered closed-loop system maintains stable operation with a reasonable tolerance to gain and phase variations.

**Fig 29:**
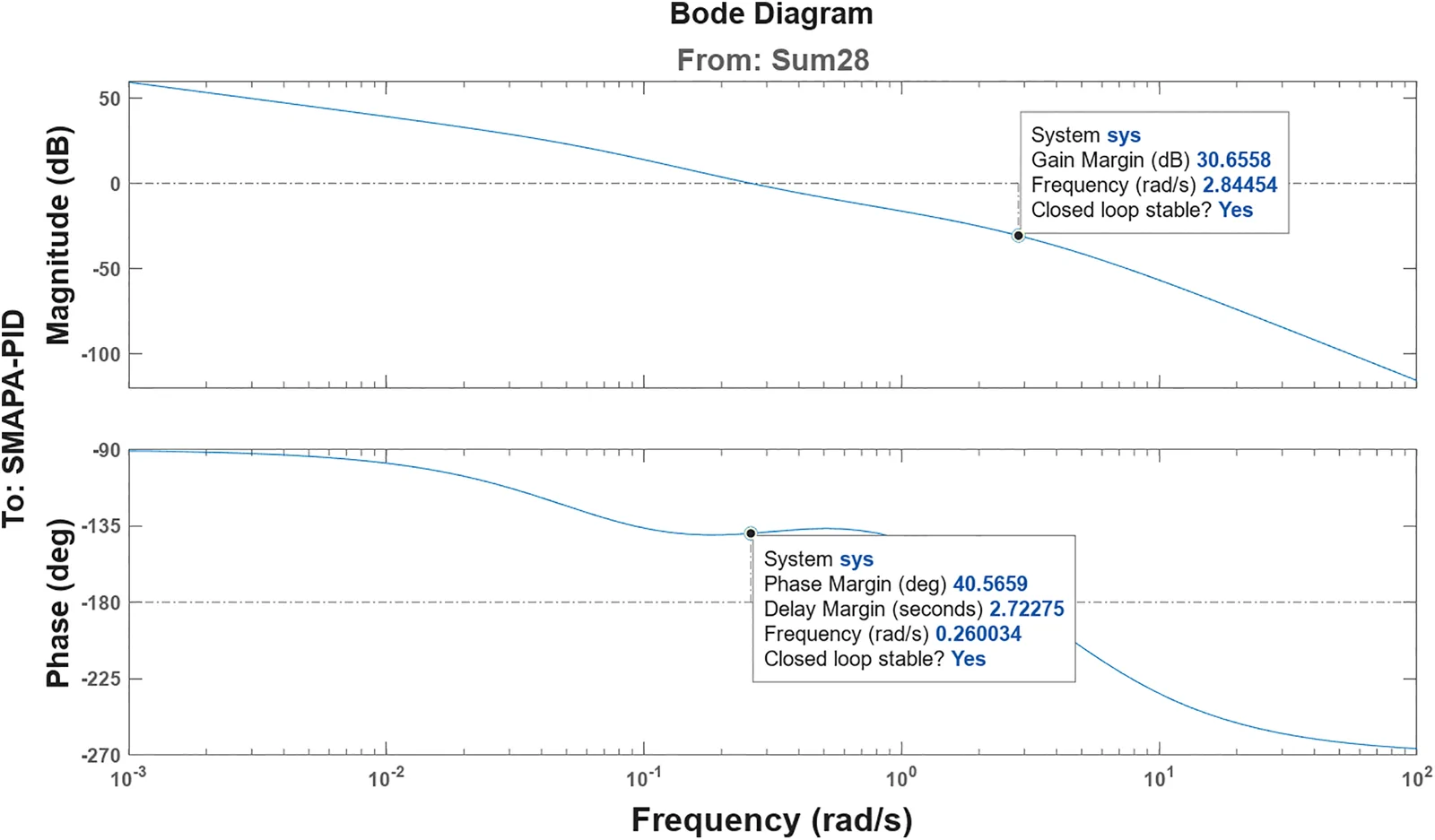
Bode plot of proposed controller.

## 6. Conclusion

The simulation results demonstrate that the proposed cascaded SMAPA–PID controller provides improved frequency regulation performance in both single-area and two-area power systems. Under step load disturbances, random load variations, and wind power fluctuations, the proposed controller achieves lower overshoot and shorter settling time compared with the considered conventional controllers. The effect of parameter uncertainty is also investigated by varying key system parameters, demonstrating the robustness of the proposed controller under changes in system operating conditions. In addition, frequency-domain stability is assessed using Bode analysis to further verify the stability characteristics of the proposed control system. For the two-area system, the proposed controller is also compared with the recently developed SPPI-based approach, providing a direct comparison with an adaptive LFC strategy. Furthermore, real-time validation is performed for the two-area system, where the proposed controller maintains satisfactory dynamic performance under practical implementation conditions. Overall, the results demonstrate that the proposed cascaded SMAPA–PID controller combines online parameter adaptation with the simplicity of the PI–PID structure, providing effective and robust frequency regulation under the considered operating conditions.

However, the present study is limited to single-area and two-area LFC systems and the considered disturbance and uncertainty scenarios. Future work will focus on extending the proposed controller to larger interconnected multi-area power systems with higher renewable-energy penetration, communication uncertainties, and cyber-physical constraints. Further investigation of the computational requirements and real-time performance under more complex operating conditions will also be considered.
