# Supplementary material for: Adaptive–based cascaded PI–PID controller for load frequency regulation with real time verification
Source: PLoS One. 2026 Nov 6;21(11):e0362396. doi: 10.1371/journal.pone.0362396 (PMC13742904; doi:10.1371/journal.pone.0362396)
Supplement: Supplementary file 1 [file pone.0362396.s002.docx]

Reviewers' comments:

Reviewer's Responses to Questions

**Comments to the Author**

**Reviewer #1:**

- The novelty of the proposed adaptive cascaded SMAPA–PI–PID controller should be highlighted more clearly by explicitly distinguishing it from recently published adaptive and cascaded LFC control strategies.

**Response:**

Thank you for this valuable comment. The novelty of the proposed adaptive cascaded SMAPA–PI–PID controller has been clarified in the revised manuscript by highlighting its main differences from existing adaptive and cascaded LFC strategies. The online SMAPA-based adaptation mechanism and its integration with the cascaded PI–PID structure are now clearly emphasized. In addition, the comparison with the recent SPPI-based approach further clarifies the contribution of the proposed method.

-The literature review requires further strengthening by including more recent studies (2023–2026) on adaptive, intelligent, and data-driven load frequency control techniques, along with a critical comparison of their limitations.

**Response:**

Thank you for this valuable comment. The literature review has been strengthened by adding recent studies from 2024–2026 on adaptive, intelligent, and data-driven LFC techniques. The limitations of the reviewed methods have also been briefly discussed to clarify the research gap and better highlight the novelty of the proposed approach.

-The mathematical formulation of the SMAPA algorithm should be explained in greater detail, including the derivation of the adaptive update law and the selection criteria for the algorithm parameters.

**Response:**
Thank you for this valuable comment. The mathematical formulation of the proposed SMAPA-based adaptive mechanism has been expanded in the revised manuscript, with the adaptive weight-update equations, controller gain adaptation, and main parameters clearly defined. The online adaptation procedure has also been described in detail to clarify how the controller parameters are updated based on the prediction error and prescribed tolerance. These additions improve the clarity, transparency, and reproducibility of the proposed adaptive control strategy.

-The controller tuning methodology is currently described as a trial-and-error process. A more systematic tuning procedure or justification should be provided to improve the reproducibility of the proposed approach.

Response:

Thank you for this valuable comment. The tuning procedure has been clarified in the revised manuscript. Instead of describing the tuning as trial-and-error, we now specify that a one-factor-at-a-time procedure was followed, where each controller coefficient was varied while the other coefficients were kept fixed, and its effect on the control performance was evaluated. The selected parameters were then fixed for all subsequent case studies to ensure consistent and reproducible results.

-The manuscript would benefit from a computational complexity analysis and discussion of the real-time implementation requirements compared with the benchmark controllers.
Although real-time validation is presented, additional information regarding the experimental platform, sampling time, hardware specifications, communication delays, and implementation constraints should be included.

Response:

Thank you for this valuable comment. A comparative analysis of the computational requirements and real-time suitability of the proposed controller and benchmark PI/PID controllers has been added in Table 14.

| Parameter | Specification |
| --- | --- |
| Real-time target platform | Speedgoat Performance Real-Time Target Machine |
| CPU | Intel® Core™ Processor, 3.1 GHz, Quad-Core |
| Real-time operating system | Simulink® Real-Time™ |
| MATLAB compatibility | R2021a and later |
| Memory | 8 GB RAM |
| Storage | 128 GB SSD |
| Simulation mode | Fixed-step discrete real-time simulation |
| I/O configuration | Analog/Digital I/O through Speedgoat I/O modules |
| Measurement device | FLUKE MDA-550 Power Analyzer |

-The robustness of the proposed controller should be further validated under parameter uncertainties, measurement noise, governor dead-band, generation rate constraints, and communication delays to better reflect practical operating conditions.

**Response:**

Thank you for this valuable comment. To address this concern, an additional parameter uncertainty case has been added to the revised manuscript. In this case, the system parameters, , and were varied by from their nominal values. The corresponding simulation results have been included in the revised manuscript to further assess the robustness of the proposed controller under parameter uncertainties.

-The comparison with existing methods should include statistical performance measures or multiple simulation runs to demonstrate that the reported improvements are consistent and statistically significant.

**Response**

Thank you for this valuable comment. To further strengthen the consistency and reliability of the reported results, the revised manuscript now includes a quantitative comparison based on the IAE performance index, a robustness analysis under ±20% variations of the main system parameters, and a frequency-domain analysis using Bode plots. In addition, the proposed cascaded SMAPA–PID controller has been compared with the SPPI controller under the same operating conditions. These additional analyses provide quantitative evidence of the performance improvement and robustness of the proposed controller across different operating conditions.

-Several grammatical errors, inconsistent terminology, and formatting issues are present throughout the manuscript. A thorough English language revision is recommended to improve readability and presentation quality.
The figures and tables should be improved by increasing their resolution, enlarging axis labels and legends, and ensuring consistent notation between the mathematical equations and simulation results.

**Response**

Thank you for your valuable comments, the grammar, terminology, formatting and English have been revised. Moreover, the quality of the figures is enhanced in the revised version of the manuscript.

-The conclusion should be expanded to discuss the practical implications, limitations of the proposed controller, and potential future research directions, such as extension to multi-area power systems with renewable energy uncertainty and cyber-physical constraints.

**Response**

Thank you for this valuable comment. The conclusion has been expanded to discuss the practical implications and limitations of the proposed controller. Future research directions, including extension to larger multi-area systems with renewable-energy uncertainty, communication uncertainties, and cyber-physical constraints, have also been added.

**Reviewer #2:**

1. The manuscript addresses an important problem in load frequency control (LFC), but the novelty of the proposed adaptive cascaded SMAPA–PI–PID controller over existing adaptive and cascaded PID approaches should be more clearly established.

**Response:**
Thank you for this valuable comment. We have revised the manuscript to clarify the novelty and contributions of the proposed adaptive cascaded SMAPA–PI–PID controller. The revised text highlights the integration of the SMAPA-based online parameter adaptation mechanism with the cascaded PID structure and clearly explains its distinction from conventional fixed-gain and existing adaptive LFC approaches. The contribution section has also been revised accordingly.

2. The mathematical formulation of the SMAPA-based adaptive mechanism should be presented in greater detail, including the parameter update equations and adaptation algorithm.

**Response:**

Thank you for this valuable comment. The mathematical formulation of the proposed SMAPA-based adaptive mechanism has been explained in greater detail, with all relevant equations and parameters clearly defined. In addition, a pseudocode and a flowchart have been added to clearly illustrate the online adaptation and parameter-update procedures, thereby improving the clarity and reproducibility of the proposed method.

The manuscript lacks theoretical stability analysis. A Lyapunov-based or equivalent stability proof would strengthen the technical contribution.

**Response:**

We thank the reviewer for this valuable suggestion. A frequency-domain stability analysis has been added to the revised manuscript using the Bode diagram to further assess the stability and robustness of the proposed cascaded SMAPA–PID controller. The corresponding analysis is presented in the newly added **“Frequency-Domain Stability Analysis”** subsection.

3. The controller should be compared with more recent state-of-the-art LFC techniques (e.g., fractional-order PID, model predictive control, sliding mode control, adaptive robust control, or reinforcement learning-based controllers) instead of only conventional PI/PID controllers. The following literature should be considered for comparison: Adaptive synthetic inertia control framework for distributed energy resources in low-inertia microgrid, doi: 10.1109/ACCESS.2022.3177661; Fractional order AGC design for power systems via artificial gorilla troops optimizer, doi:10.1109/PEDES56012.2022.10079975;Coordinated Synthetic Inertia Control Provision from Distributed Energy Resources and Energy Storage Systems, doi:10.1109/ICICCSP53532.2022.9862397.

**Response:**

Thank you for this valuable comment. To address this concern, a new case study involving random load variations has been added to the revised manuscript. The proposed SMAPA–PID controller has been compared with the recently published SPPI-based controller under the same operating conditions. The corresponding comparison results and transient performance metrics have been included in the revised manuscript.

4. The fairness of the comparative study should be clarified by specifying whether all benchmark controllers were tuned using identical objective functions, constraints, and operating conditions.

**Response:**
 Thank you for this valuable comment. To ensure a fair comparison, the proposed SMAPA–PID controller and the benchmark PI and PID controllers were evaluated under the same operating conditions using the Integral Absolute Error (IAE) as a common performance index. The revised manuscript has been updated to clarify the comparison procedure and the performance evaluation criteria.

5. The manuscript claims applicability to large-scale interconnected power systems; however, only single-area and two-area systems are investigated. These claims should either be validated through additional case studies or appropriately moderated. The scalability of the proposed controller should be demonstrated using multi-area systems with more than two interconnected control areas.

**Response:**

However, the present study is limited to single-area and two-area interconnected LFC systems. The scalability of the proposed controller to larger multi-area power systems with more than two interconnected control areas has not been investigated in the present work. This aspect will be considered in future research to further assess the scalability of the proposed adaptive control strategy.

6. PLOS authors have the option to publish the peer review history of their article ([what does this mean?](https://track.editorialmanager.com/CL0/https:%2F%2Fjournals.plos.org%2Fplosone%2Fs%2Feditorial-and-peer-review-process%23loc-peer-review-history/1/010f019fca9cb114-0fe7ba24-5d1c-445a-9b12-e549ffa63d84-000000/C-JsT7JwbdNMdGuv0Q3UYR99HWC7I8QAZkKRVggjqNM=258)). If published, this will include your full peer review and any attached files.


If you choose “no”, your identity will remain anonymous but your review may still be made public.

**Do you want your identity to be public for this peer review?** For information about this choice, including consent withdrawal, please see our [Privacy Policy](https://track.editorialmanager.com/CL0/https:%2F%2Fwww.plos.org%2Fprivacy-policy/1/010f019fca9cb114-0fe7ba24-5d1c-445a-9b12-e549ffa63d84-000000/IzOhXnMDdtimMjARUi9KgJ-oa9WYqV9XLo-VW5CVsv0=258).

Reviewer #1: **Yes:** Dr. Vineet Kumar

Reviewer #2: No

[NOTE: If reviewer comments were submitted as an attachment file, they will be attached to this email and accessible via the submission site. Please log into your account, locate the manuscript record, and check for the action link "View Attachments". If this link does not appear, there are no attachment files.]

To ensure your figures meet our technical requirements, please review our figure guidelines: https://journals.plos.org/plosone/s/figures

You may also use PLOS’s free figure tool, NAAS, to help you prepare publication quality figures: https://journals.plos.org/plosone/s/figures#loc-tools-for-figure-preparation.

NAAS will assess whether your figures meet our technical requirements by comparing each figure against our figure specifications.
