# Supplementary material for: Adaptive–based cascaded PI–PID controller for load frequency regulation with real time verification
Source: PLoS One. 2026 Nov 6;21(11):e0362396. doi: 10.1371/journal.pone.0362396 (PMC13742904; doi:10.1371/journal.pone.0362396)
Supplement: Peer review history

# Peer review: Decision Letter 0

**DOI:** 10.1371/journal.pone.0362396.r001

**Authors:** Vineet Kumar

3 Aug 2026

Dear Dr. EL-Ebiary,

Thank you for submitting your manuscript to PLOS ONE. After careful consideration, we feel that it has merit but does not fully meet PLOS ONE’s publication criteria as it currently stands. Therefore, we invite you to submit a revised version of the manuscript that addresses the points raised during the review process.

Please submit your revised manuscript by Oct 02 2026 11:59PM. If you will need more time than this to complete your revisions, please reply to this message or contact the journal office at plosone@plos.org. When you're ready to submit your revision, log on to https://www.editorialmanager.com/pone/ and select the 'Submissions Needing Revision' folder to locate your manuscript file.

- A letter that responds to each point raised by the academic editor and reviewer(s). You should upload this letter as a separate file labeled 'Response to Reviewers'.
- A marked-up copy of your manuscript that highlights changes made to the original version. You should upload this as a separate file labeled 'Revised Manuscript with Track Changes'.
- An unmarked version of your revised paper without tracked changes. You should upload this as a separate file labeled 'Manuscript'.

If you would like to make changes to your financial disclosure, please include your updated statement in your cover letter. Guidelines for resubmitting your figure files are available below the reviewer comments at the end of this letter.

If applicable, we recommend that you deposit your laboratory protocols in protocols.io to enhance the reproducibility of your results. Protocols.io assigns your protocol its own identifier (DOI) so that it can be cited independently in the future. For instructions see: https://journals.plos.org/plosone/s/submission-guidelines#loc-laboratory-protocols. Additionally, PLOS ONE offers an option for publishing peer-reviewed Lab Protocol articles, which describe protocols hosted on protocols.io. Read more information on sharing protocols at https://plos.org/protocols?utm_medium=editorial-email&utm_source=authorletters&utm_campaign=protocols.

As the corresponding author, your ORCID iD is verified in the submission system and will appear in the published article. PLOS supports the use of ORCID, and we encourage all coauthors to register for an ORCID iD and use it as well. Please encourage your coauthors to verify their ORCID iD within the submission system before final acceptance, as unverified ORCID iDs will not appear in the published article. *Only*  the individual author can complete the verification step; PLOS staff *cannot*  verify ORCID iDs on behalf of authors.

We look forward to receiving your revised manuscript.

Kind regards,

Vineet Kumar

Academic Editor

PLOS One

Journal Requirements:

When submitting your revision, we need you to address these additional requirements.

1. Please ensure that your manuscript meets PLOS ONE's style requirements, including those for file naming. The PLOS ONE style templates can be found at

https://journals.plos.org/plosone/s/file?id=wjVg/PLOSOne_formatting_sample_main_body.pdf and

https://journals.plos.org/plosone/s/file?id=ba62/PLOSOne_formatting_sample_title_authors_affiliations.pdf

2. Please note that PLOS One has specific guidelines on code sharing for submissions in which author-generated code underpins the findings in the manuscript. In these cases, we expect all author-generated code to be made available without restrictions upon publication of the work. Please review our guidelines at https://journals.plos.org/plosone/s/materials-and-software-sharing#loc-sharing-code and ensure that your code is shared in a way that follows best practice and facilitates reproducibility and reuse.

3. We note that the grant information you provided in the ‘Funding Information’ and ‘Financial Disclosure’ sections do not match.

When you resubmit, please ensure that you provide the correct grant numbers for the awards you received for your study in the ‘Funding Information’ section.

4. Thank you for stating the following financial disclosure:

“Prince Sattam Bin Abdulaziz University Project”

Please state what role the funders took in the study.  If the funders had no role, please state: "The funders had no role in study design, data collection and analysis, decision to publish, or preparation of the manuscript."

If this statement is not correct you must amend it as needed.

Please include this amended Role of Funder statement in your cover letter; we will change the online submission form on your behalf.

5. Thank you for stating the following financial disclosure:

“Prince Sattam Bin Abdulaziz University Project”

We note that one or more of the authors is affiliated with the funding organization, indicating the funder may have had some role in the design, data collection, analysis or preparation of your manuscript for publication; in other words, the funder played an indirect role through the participation of the co-authors. If the funding organization did not play a role in the study design, data collection and analysis, decision to publish, or preparation of the manuscript and only provided financial support in the form of authors' salaries and/or research materials, please do the following:

1. Review your statements relating to the author contributions, and ensure you have specifically and accurately indicated the role(s) that these authors had in your study. These amendments should be made in the online form.

2. Confirm in your cover letter that you agree with the following statement, and we will change the online submission form on your behalf:

“The funder provided support in the form of salaries for authors [insert relevant initials], but did not have any additional role in the study design, data collection and analysis, decision to publish, or preparation of the manuscript. The specific roles of these authors are articulated in the ‘author contributions’ section.

6. In the online submission form you indicate that your data is not available for proprietary reasons and have provided a contact point for accessing this data. Please note that your current contact point is a co-author on this manuscript. According to our Data Policy, the contact point must not be an author on the manuscript and must be an institutional contact, ideally not an individual. Please revise your data statement to a non-author institutional point of contact, such as a data access or ethics committee, and send this to us via return email. Please also include contact information for the third party organization, and please include the full citation of where the data can be found.

7. We note that Figure 21 in your submission contain copyrighted images. All PLOS content is published under the Creative Commons Attribution License (CC BY 4.0), which means that the manuscript, images, and Supporting Information files will be freely available online, and any third party is permitted to access, download, copy, distribute, and use these materials in any way, even commercially, with proper attribution. For more information, see our copyright guidelines: http://journals.plos.org/plosone/s/licenses-and-copyright.

We require you to either (1) present written permission from the copyright holder to publish these figures specifically under the CC BY 4.0 license, or (2) remove the figures from your submission:

1. You may seek permission from the original copyright holder of Figure(s) [#] to publish the content specifically under the CC BY 4.0 license.

We recommend that you contact the original copyright holder with the Content Permission Form (http://journals.plos.org/plosone/s/file?id=7c09/content-permission-form.pdf) and the following text:

“I request permission for the open-access journal PLOS ONE to publish XXX under the Creative Commons Attribution License (CCAL) CC BY 4.0 (http://creativecommons.org/licenses/by/4.0/). Please be aware that this license allows unrestricted use and distribution, even commercially, by third parties. Please reply and provide explicit written permission to publish XXX under a CC BY license and complete the attached form.”

Please upload the completed Content Permission Form or other proof of granted permissions as an "Other" file with your submission.

In the figure caption of the copyrighted figure, please include the following text: “Reprinted from [ref] under a CC BY license, with permission from [name of publisher], original copyright [original copyright year].”

2. If you are unable to obtain permission from the original copyright holder to publish these figures under the CC BY 4.0 license or if the copyright holder’s requirements are incompatible with the CC BY 4.0 license, please either i) remove the figure or ii) supply a replacement figure that complies with the CC BY 4.0 license. Please check copyright information on all replacement figures and update the figure caption with source information. If applicable, please specify in the figure caption text when a figure is similar but not identical to the original image and is therefore for illustrative purposes only.

8. If the reviewer comments include a recommendation to cite specific previously published works, please review and evaluate these publications to determine whether they are relevant and should be cited. There is no requirement to cite these works unless the editor has indicated otherwise.

Additional Editor Comments:

Based on the reviewers reports, the manuscript has been recommended for major revision. The authors are required to thoroughly revise the manuscript by carefully addressing all the comments and concerns raised by the reviewers. In particular, the literature review should be strengthened to clearly establish the novelty and research gap, and the proposed methodology should be explained in greater detail with appropriate theoretical justification. The experimental results should be expanded and compared with recent state-of-the-art studies to validate the effectiveness and significance of the proposed approach. In addition, the discussion of the results should be improved to highlight the practical implications, advantages, and limitations of the work. The manuscript should also be carefully checked for grammatical errors, consistency in terminology, formatting issues, and the quality of figures and tables. The authors are encouraged to prepare a detailed point-by-point response explaining how each reviewer comment has been addressed. A comprehensive revision addressing these aspects is essential before the manuscript can be reconsidered for publication.

[Note: HTML markup is below. Please do not edit.]

Reviewers' comments:

Reviewer's Responses to Questions

**Comments to the Author**

1. Is the manuscript technically sound, and do the data support the conclusions?

Reviewer #1: Partly

Reviewer #2: Yes

**********

2. Has the statistical analysis been performed appropriately and rigorously?

Reviewer #1: Yes

Reviewer #2: N/A

**********

3. Have the authors made all data underlying the findings in their manuscript fully available?

The PLOS Data policy

Reviewer #1: Yes

Reviewer #2: Yes

**********

4. Is the manuscript presented in an intelligible fashion and written in standard English?

Reviewer #1: Yes

Reviewer #2: Yes

**********

Reviewer #1: The novelty of the proposed adaptive cascaded SMAPA–PI–PID controller should be highlighted more clearly by explicitly distinguishing it from recently published adaptive and cascaded LFC control strategies.

The literature review requires further strengthening by including more recent studies (2023–2026) on adaptive, intelligent, and data-driven load frequency control techniques, along with a critical comparison of their limitations.

The mathematical formulation of the SMAPA algorithm should be explained in greater detail, including the derivation of the adaptive update law and the selection criteria for the algorithm parameters.

The controller tuning methodology is currently described as a trial-and-error process. A more systematic tuning procedure or justification should be provided to improve the reproducibility of the proposed approach.

The manuscript would benefit from a computational complexity analysis and discussion of the real-time implementation requirements compared with the benchmark controllers.

Although real-time validation is presented, additional information regarding the experimental platform, sampling time, hardware specifications, communication delays, and implementation constraints should be included.

The robustness of the proposed controller should be further validated under parameter uncertainties, measurement noise, governor dead-band, generation rate constraints, and communication delays to better reflect practical operating conditions.

The comparison with existing methods should include statistical performance measures or multiple simulation runs to demonstrate that the reported improvements are consistent and statistically significant.

Several grammatical errors, inconsistent terminology, and formatting issues are present throughout the manuscript. A thorough English language revision is recommended to improve readability and presentation quality.

The figures and tables should be improved by increasing their resolution, enlarging axis labels and legends, and ensuring consistent notation between the mathematical equations and simulation results.

The conclusion should be expanded to discuss the practical implications, limitations of the proposed controller, and potential future research directions, such as extension to multi-area power systems with renewable energy uncertainty and cyber-physical constraints.

Reviewer #2: 1. The manuscript addresses an important problem in load frequency control (LFC), but the novelty of the proposed adaptive cascaded SMAPA–PI–PID controller over existing adaptive and cascaded PID approaches should be more clearly established.

2. The mathematical formulation of the SMAPA-based adaptive mechanism should be presented in greater detail, including the parameter update equations and adaptation algorithm.

The manuscript lacks a theoretical stability analysis. A Lyapunov-based or equivalent stability proof would strengthen the technical contribution.

3. The controller should be compared with more recent state-of-the-art LFC techniques (e.g., fractional-order PID, model predictive control, sliding mode control, adaptive robust control, or reinforcement learning-based controllers) instead of only conventional PI/PID controllers. The following literature should be considered for comparison: Adaptive synthetic inertia control framework for distributed energy resources in low-inertia microgrid, doi: 10.1109/ACCESS.2022.3177661; Fractional order AGC design for power systems via artificial gorilla troops optimizer, doi:10.1109/PEDES56012.2022.10079975;Coordinated Synthetic Inertia Control Provision from Distributed Energy Resources and Energy Storage Systems, doi:10.1109/ICICCSP53532.2022.9862397.

4. The fairness of the comparative study should be clarified by specifying whether all benchmark controllers were tuned using identical objective functions, constraints, and operating conditions.

5. The manuscript claims applicability to large-scale interconnected power systems; however, only single-area and two-area systems are investigated. These claims should either be validated through additional case studies or appropriately moderated. The scalability of the proposed controller should be demonstrated using multi-area systems with more than two interconnected control areas.

**********

what does this mean?). If published, this will include your full peer review and any attached files.

If you choose “no”, your identity will remain anonymous but your review may still be made public.

**Do you want your identity to be public for this peer review?**  For information about this choice, including consent withdrawal, please see our Privacy Policy

Reviewer #1: **Yes:**  Dr. Vineet Kumar

Reviewer #2: No

**********

[NOTE: If reviewer comments were submitted as an attachment file, they will be attached to this email and accessible via the submission site. Please log into your account, locate the manuscript record, and check for the action link "View Attachments". If this link does not appear, there are no attachment files.]

To ensure your figures meet our technical requirements, please review our figure guidelines: https://journals.plos.org/plosone/s/figures

You may also use PLOS’s free figure tool, NAAS, to help you prepare publication quality figures: https://journals.plos.org/plosone/s/figures#loc-tools-for-figure-preparation.

NAAS will assess whether your figures meet our technical requirements by comparing each figure against our figure specifications.

# Author comment: Author response to Decision Letter 1

**DOI:** 10.1371/journal.pone.0362396.r002

18 Oct 2026

Thank you for your valuable comments that helped to enhance the quality of the manuscript. The response to the reviewer comments is in the response word file.

Attachment Submitted filename: Response to Reviewers.docx

# Peer review: Decision Letter 1

**DOI:** 10.1371/journal.pone.0362396.r003

**Authors:** Vineet Kumar

25 Oct 2026

Adaptive–Based Cascaded PI–PID Controller for Load Frequency Regulation with Real Time Verification

PONE-D-26-35307R1

Dear Dr. EL-Ebiary,

We’re pleased to inform you that your manuscript has been judged scientifically suitable for publication and will be formally accepted for publication once it meets all outstanding technical requirements.

Within one week, you’ll receive an e-mail detailing the required amendments. When these have been addressed, you’ll receive a formal acceptance letter and your manuscript will be scheduled for publication.

An invoice will be generated when your article is formally accepted. Please note, if your institution has a publishing partnership with PLOS and your article meets the relevant criteria, all or part of your publication costs will be covered. Please make sure your user information is up-to-date by logging into Editorial Manager at Editorial Manager® and clicking the ‘Update My Information' link at the top of the page. For questions related to billing, please contact billing support.

If your institution or institutions have a press office, please notify them about your upcoming paper to help maximize its impact. If they’ll be preparing press materials, please inform our press team as soon as possible -- no later than 48 hours after receiving the formal acceptance. Your manuscript will remain under strict press embargo until 2 pm Eastern Time on the date of publication. For more information, please contact onepress@plos.org.

Kind regards,

Vineet Kumar

Academic Editor

PLOS One

Additional Editor Comments (optional):

The paper is accepted in its present form and requires no further revision.

Reviewers' comments:

Reviewer's Responses to Questions

**Comments to the Author**

Reviewer #1: All comments have been addressed

Reviewer #2: All comments have been addressed

**********

2. Is the manuscript technically sound, and do the data support the conclusions?

Reviewer #1: Yes

Reviewer #2: Yes

**********

3. Has the statistical analysis been performed appropriately and rigorously?

Reviewer #1: Yes

Reviewer #2: N/A

**********

4. Have the authors made all data underlying the findings in their manuscript fully available?

The PLOS Data policy

Reviewer #1: Yes

Reviewer #2: Yes

**********

5. Is the manuscript presented in an intelligible fashion and written in standard English?

Reviewer #1: Yes

Reviewer #2: Yes

**********

Reviewer #1: Authors have done well to address all the queries raised in earlier versions... I recommend publication

Reviewer #2: The authors have adequately addressed the concerns raised by this reviewer. I don't have any further comments.

**********

what does this mean?). If published, this will include your full peer review and any attached files.

If you choose “no”, your identity will remain anonymous but your review may still be made public.

**Do you want your identity to be public for this peer review?**  For information about this choice, including consent withdrawal, please see our Privacy Policy

Reviewer #1: **Yes:**  Vineet Kumar

Reviewer #2: No

**********

# Editor report: Acceptance letter

**DOI:** 10.1371/journal.pone.0362396.r004

**Authors:** Vineet Kumar

PONE-D-26-35307R1

PLOS One

Dear Dr. EL-Ebiary,

I'm pleased to inform you that your manuscript has been deemed suitable for publication in PLOS One. Congratulations! Your manuscript is now being handed over to our production team.

At this stage, our production department will prepare your paper for publication. This includes ensuring the following:

* All references, tables, and figures are properly cited

* All relevant supporting information is included in the manuscript submission,

* There are no issues that prevent the paper from being properly typeset

You will receive further instructions from the production team, including instructions on how to review your proof when it is ready. Please keep in mind that we are working through a large volume of accepted articles, so please give us a few days to review your paper and let you know the next and final steps.

Lastly, if your institution or institutions have a press office, please let them know about your upcoming paper now to help maximize its impact. If they'll be preparing press materials, please inform our press team within the next 48 hours. Your manuscript will remain under strict press embargo until 2 pm Eastern Time on the date of publication. For more information, please contact onepress@plos.org.

You will receive an invoice from PLOS for your publication fee after your manuscript has reached the completed accept phase. If you receive an email requesting payment before acceptance or for any other service, this may be a phishing scheme. Learn how to identify phishing emails and protect your accounts at https://explore.plos.org/phishing.

If we can help with anything else, please email us at customercare@plos.org.

Thank you for submitting your work to PLOS One and supporting open access.

Kind regards,

PLOS One Editorial Office Staff

on behalf of

Dr. Vineet Kumar

Academic Editor

PLOS One
